# Characterisation of sequence–structure–function space in sensor–effector integrators of phytochrome-regulated diguanylate cyclases

**DOI:** 10.1007/s43630-022-00255-7

**Published:** 2022-07-05

**Authors:** Cornelia Böhm, Geoffrey Gourinchas, Sophie Zweytick, Elvira Hujdur, Martina Reiter, Sara Trstenjak, Christoph Wilhelm Sensen, Andreas Winkler

**Affiliations:** 1 Institute of Biochemistry, Graz University of Technology, 8010 Graz, Austria; 2 BioTechMed-Graz, 8010 Graz, Austria; 3 Department of Integrated Structural Biology, Institut de Génétique et de Biologie Moléculaire et Cellulaire (IGBMC), 67404 Illkirch, France; 4 Hungarian Centre of Excellence for Molecular Medicine, Római körút 21, 6723 Szeged, Hungary

**Keywords:** Bacteriophytochrome, Coiled-coil linker, GGDEF, Photoreceptor, Phylogenetic analysis, Sequence similarity network

## Abstract

Understanding the relationship between protein sequence, structure and function is one of the fundamental challenges in biochemistry. A direct correlation, however, is often not trivial since protein dynamics also play an important functional role—especially in signal transduction processes. In a subfamily of bacterial light sensors, phytochrome-activated diguanylate cyclases (PadCs), a characteristic coiled-coil linker element connects photoreceptor and output module, playing an essential role in signal integration. Combining phylogenetic analyses with biochemical characterisations, we were able to show that length and composition of this linker determine sensor–effector function and as such are under considerable evolutionary pressure. The linker length, together with the upstream PHY-specific domain, influences the dynamic range of effector activation and can even cause light-induced enzyme inhibition. We demonstrate phylogenetic clustering according to linker length, and the development of new linker lengths as well as new protein function within linker families. The biochemical characterisation of PadC homologs revealed that the functional coupling of PHY dimer interface and linker element defines signal integration and regulation of output functionality. A small subfamily of PadCs, characterised by a linker length breaking the coiled-coil pattern, shows a markedly different behaviour from other homologs. The effect of the central helical spine on PadC function highlights its essential role in signal integration as well as direct regulation of diguanylate cyclase activity. Appreciation of sensor–effector linkers as integrator elements and their coevolution with sensory modules is a further step towards the use of functionally diverse homologs as building blocks for rationally designed optogenetic tools.

**Graphical abstract:**

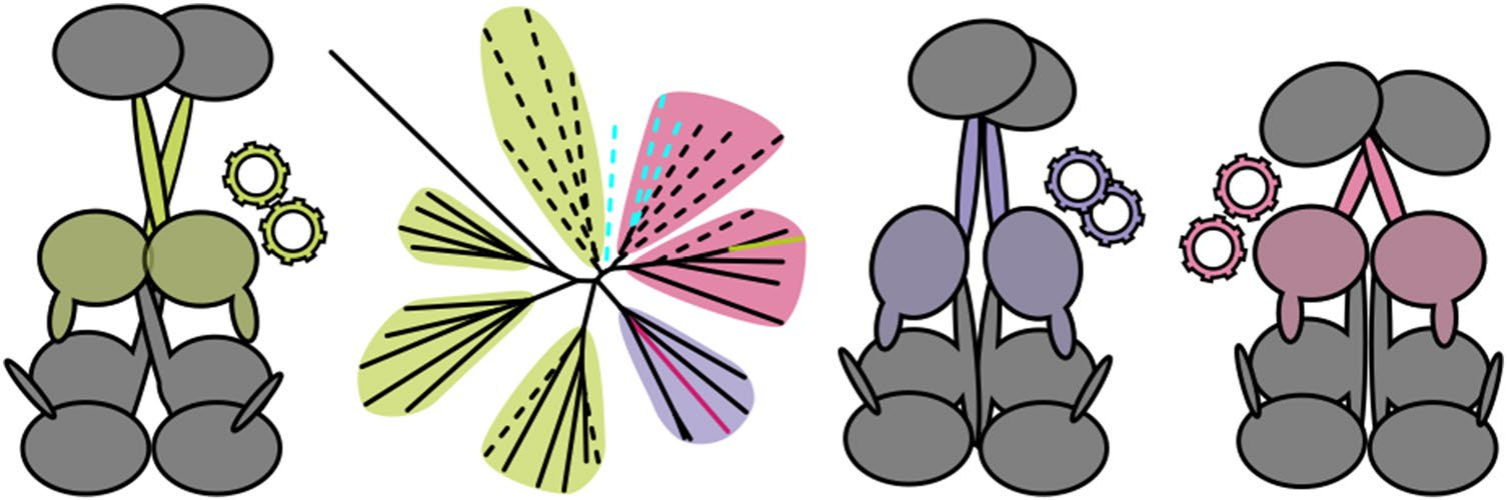

**Supplementary Information:**

The online version contains supplementary material available at 10.1007/s43630-022-00255-7.

## Introduction

Protein function depends strongly on protein structure; and the three-dimensional arrangement of amino acids is tightly linked to their sequence. Conservation of protein structure is, however, considerably higher than that of the amino acid sequence [1] and even sequences with no evolutionary relationship can sometimes fold into the same three-dimensional architecture. While similar sequences often imply functional similarities, the situation is further complicated by proteins of different structures performing analogous functions [2]. This complex interplay of sequence, structure and function is one of the fundamental problems in functional annotation of proteins, since the degree of conservation of sequence- and structural features that are essential for different functionalities also provides nature with mechanisms to modulate biological properties and even develop novel activities. While important on a general protein evolution level, this flexibility of sequence–structure–function space is also central to the development of functional sensor–effector systems where domains sensing environmental signals are covalently linked to biological effector units, thereby enabling regulation of enzymatic activities, gene transcription or biological interactions.

**Fig. 1:**
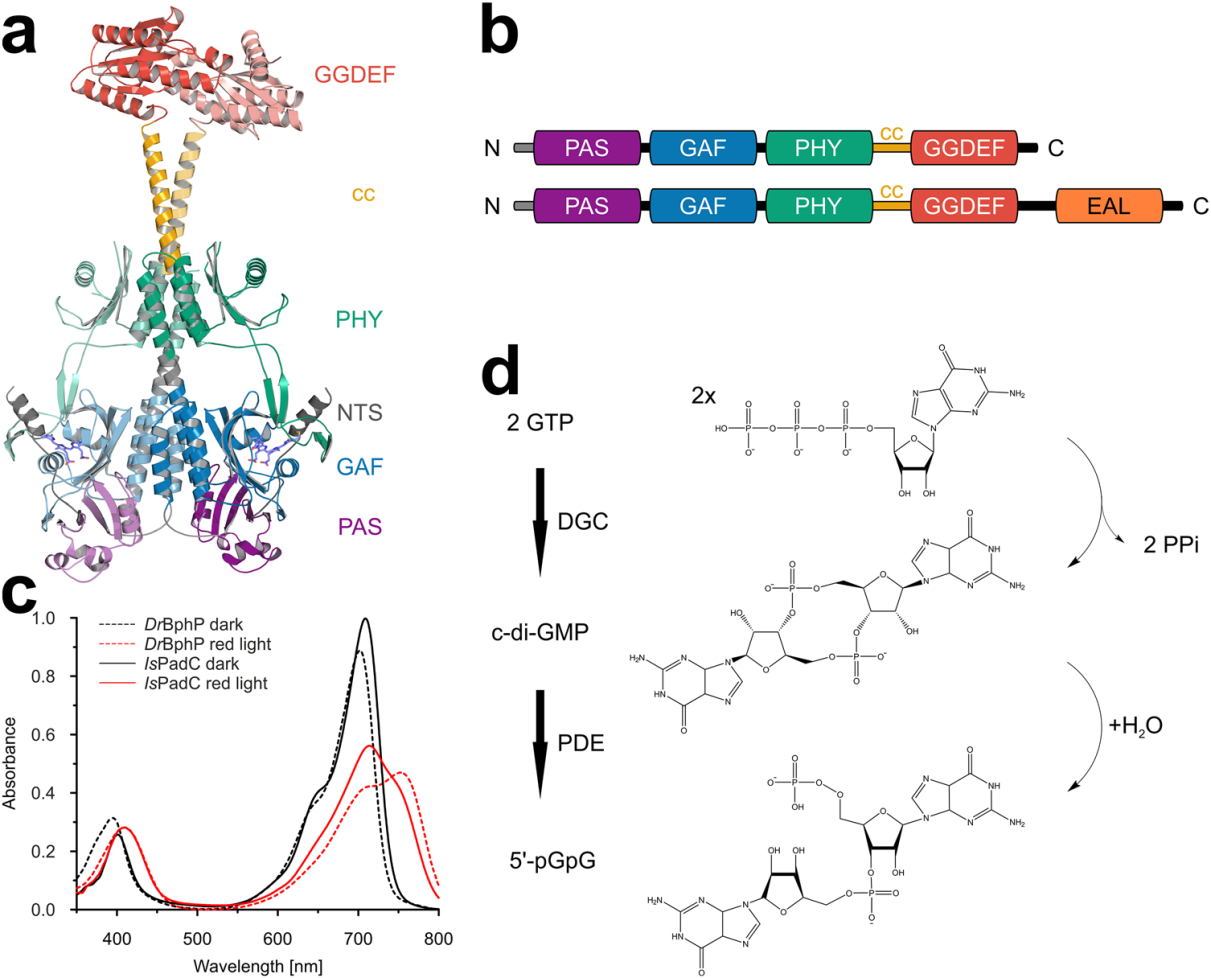
Phytochrome-activated diguanylate cyclases (PadCs). **a** Dark-state crystal structure of full-length *Is*PadC (pdb 5LLW) represented as cartoon coloured by structural domains [5]. **b** Domain architecture of PadCs and PadC-EALs, coloration of domains as presented in **panel a**. N-terminal segment (NTS) = grey; Period/ARNT/Single-minded (PAS) domain = purple; cGMP phosphodiesterase-Adenylate cyclase-FhlA (GAF) domain = blue; phytochrome-specific (PHY) domain = green; coiled-coil (cc) linker = yellow; GGDEF domain = red; EAL domain = orange. **c** Comparison of *Deinococcus radiodurans* bacteriophytochrome (*Dr*BphP, dashed) and *Is*PadC (continuous) UV/Vis spectra under non-actinic (black) and red light (red) conditions. **d** Catalytic activity of PadCs and PadC-EALs—diguanylate cyclases (DGCs) catalyse turnover of two molecules of GTP to cyclic dimeric GMP (c-di-GMP), which is further hydrolysed to 5′-pGpG [22] by the EAL phosphodiesterase domain (EAL-PDE)

One important environmental signal, light, can be sensed by a variety of photoreceptors [3] and frequently these sensors are covalently tethered to their cognate effector domains. In red light sensing bacteriophytochromes, this modularity is quite remarkable [4], but the sequence–function relationship of the corresponding linker elements is generally not well understood. For one bacteriophytochrome subfamily, the linker element has evolved as a central player in modulating bacterial second messenger production. Phytochrome-activated diguanylate cyclases (PadCs) are photoreceptors capable of reacting to red and far red light stimuli, typically resulting in the up- and downregulation of cyclic-dimeric-GMP (c-di-GMP) catalysis by the diguanylate cyclase effector [5–8]. The PadC architecture follows a classical bacteriophytochrome photosensory module of a PAS-GAF-PHY (Period/ARNT/Single-minded—cGMP phosphodiesterase/Adenylate cyclase/FhlA—PHYtochrome-specific) [9] core connected to a GGDEF output domain (Fig. 1a, b). The cofactor binding pocket located in the GAF domain binds the biliverdin IXα chromophore, isomerisation of which from its *ZZZssa* to *ZZEssa* configuration induces extensive structural rearrangements in the cofactor environment of bacteriophytochromes [4]. The PHY domain stabilises light-modulated state conformation via its tongue element in an interplay with the N-terminal segment (NTS) [5, 7, 10], which features a strictly conserved biliverdin-binding cysteine residue. GGDEF domains possess diguanylate cyclase (DGC) activity [11] condensing two molecules of GTP to form the bacterial second messenger c-di-GMP [12, 13]. Several PadCs have been found to C-terminally feature an additional EAL domain, as exemplified by the first characterised member of this family, BphG1 from *R. sphaeroides*, described by Tarutina et al. [14]. The EAL domain provides c-di-GMP phosphodiesterase (EAL-PDE) activity that further hydrolyses the DGC product to the linear dimeric GMP nucleotide 5′-pGpG [15] (Fig. 1d). GGDEF and EAL domains are often found in tandem, linked to various sensory modules, with one of the two effector domains catalytically inactive in many cases [12]; however, bi-functional GGDEF-EAL complexes have been identified as well [16, 17]. The importance of c-di-GMP as a second messenger involved in various biological processes is highlighted by the tight enzymatic control of its formation and degradation [18, 19], affecting bacterial lifestyle (including motility, biofilm-formation and differentiation) on a transcriptional, posttranscriptional and posttranslational level [12]. Even 5’-pGpG has been discussed as a potential second messenger [12, 20], being a member of the nanoRNA class of molecules, which often play a role in the control of gene expression [21].

The importance of c-di-GMP implies the necessity of precise systems to regulate cellular levels of this second messenger in response to various environmental stimuli. Signal transduction in sensory proteins allowing for such regulation is often a dynamic and intricate process [23]. In an attempt to understand these mechanisms of signal integration on a molecular level, we have recently focussed on the characterisation of PadCs with regard to sequence, structure and function.

One PadC member that has been studied in particular detail is *Is*PadC, the homolog from *Idiomarina* sp. A28L [5–7]. The crystal structure of the dark state full-length wildtype *Is*PadC (pdb 5LLW, [5]), as depicted in Fig. 1a, demonstrates the homo-dimeric arrangement which is typical for bacteriophytochromes (BphPs) and DGCs. The active site is formed at the dimer interface of the GGDEF domains [24], and the α-helical sensor–effector linker element features a coiled-coil arrangement. *Is*PadC is considered a prototypical PadC since red light-stimuli lead to pronounced upregulation of DGC activity. Biochemical characterisation of *Is*PadC has revealed a non-canonical spectral behaviour, which features a red light illuminated spectrum that prominently differs from that of several other well-characterised bacteriophytochromes such as *Deinococcus radiodurans* BphP [25] (Fig. 1c) or Agp1 [26]. In the latter cases, both the P_r_ dark state (phytochrome absorbing in red light) and the P_fr_ conformation (phytochrome absorbing in far red light) formed upon red light activation predominantly contribute to the absorption spectra acquired under the corresponding light regimes. *Is*PadC, however, has been shown to not fully occupy a canonical P_fr_ state, even though the chromophore was confirmed to be fully shifted to its 15*E* isomer. In the crystal structure of a constitutively active variant (pdb 6ET7, *Is*PadC S505V A526V [6]), only one of two PHY tongues is rearranged from β-hairpin to α-helix as observed for both extensions in other bacteriophytochromes [27], leading to the conclusion that the non-canonical light spectrum observed for wild type *Is*PadC might be attributed to differences in the biliverdin environments of the individual protomers. The mechanism of light regulation in *Is*PadC was shown to be based on local rearrangements in the cofactor environment after chromophore isomerisation including the GAF domain binding pocket, as well as the NTS. The interplay of the biliverdin isomerisation state and the altered NTS orientation with the PHY tongue translates into structural and conformational dynamic changes at the PHY domain dimer interface and consequently the central helical spine and the sensor–effector linker helices. The sensor–effector linker undergoes a translational movement corresponding to reorientation from inhibiting to stimulating coiled-coil register, enabling additional productive encounters of the effector domains and hence increased enzymatic activity [5, 6].

A second homolog that attracted attention due to its fundamental differences to *Is*PadC is *Mp*PadC (found in the organism *Marinobacter persicus*). *Mp*PadC shows barely any red light-induced upregulation of enzymatic activity and it features a canonical bacteriophytochrome light state fully populating P_fr_. On a sequence level a major discrepancy in linker length, i.e. the distance between PHY and GGDEF domains, was detected. For most PadCs, differences in linker length are multiples of seven amino acids, which is indicative of heptad repeats essential for coiled-coil formation. Indeed, in *Is*PadC the linker sequence follows a classical h-p-p-h-p-p-p (hydrophobic; polar) pattern, conventionally denoted as *a b c d e f g* [28]. Importantly, two overlapping heptad repeat patterns can formally be defined for many PadC homologs, which presumably allows for the register-switching necessary for profound upregulation of DGC activity [6]. *Mp*PadC, in contrast, features a linker length that is five residues shorter in comparison, suggesting that continuous coiled-coil interactions are impeded in this protein [8]. A series of *IsMp*PadC chimaeras revealed that the sequence of *Mp*PadC retains the ability for red light-based regulation of enzymatic activity if the missing residues are inserted, and that the PHY dimer interface plays an essential role in signal integration, which even overrides the effect of a functional linker length [8].

To understand how these drastically different functional properties are defined on a molecular level, we characterised these phytochrome-based DGCs more extensively. We performed a phylogenetic analysis of PadCs and PadC-EALs, as well as the biochemical characterisation of representative PadC homologs, which provided novel insights into the evolution as well as the importance of the central signal integration unit composed of the linker element and the PHY dimer interface. In addition, we have addressed the evolutionary emergence of different bacteriophytochrome-effector combinations to better understand the mechanisms underlying PadC sequence conservation and to get closer to answering the question of how different structural features affect overall protein function in these sensor–effector systems.

## Results

**Fig. 2:**
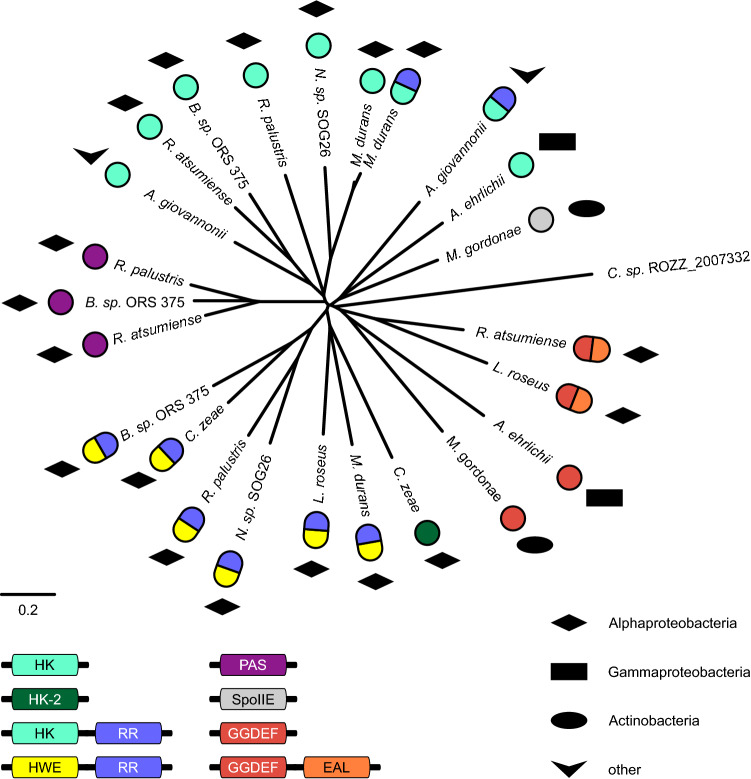
Distance tree of representative bacteriophytochromes with more than one PAS-GAF-PHY-linked output domain per organism, the cryptophyte phytochrome from *Chroomonas* sp. ROZZ_2007332 serves as outgroup. Homologous sequences tend to cluster more according to effector (coloured beads) than to evolutionary lineage (filled symbols). Histidine kinases (HK and HK-2) = turquoise and green, respectively; response regulator (RR) = blue; HWE histidine kinase = yellow; GGDEF/diguanylate cyclases = red; EAL/phosphodiesterase = orange; PAS = purple; Bacillus subtilis stage II sporulation protein E (SpoIIE)/PPM-type phosphatase domain = grey. Black shapes represent taxonomy class

The importance of reacting to the presence or absence of (red) light is reflected in the diversity of systems sensing different qualities of light and integrating this information in various signalling pathways. Bacteriophytochromes can be linked to different effector domains, which permit not only the regulation of c-di-GMP levels but also control of auto-phosphorylation or phosphatase activities, amongst other reactions. Interestingly, several bacterial organisms feature proteins with more than one effector domain linked to a phytochrome-based sensory module. Phylogenetic relationships between representatives of such PAS-GAF-PHY architectures with different effectors present in one organism are represented in Fig. 2. For *Rhodopseudomonas palustris*, for example, database entries exist for bacteriophytochromes linked to a PAS domain, a histidine kinase, a HWE histidine kinase with an attached response regulator, and even the sensory module without any output domain (not depicted in the distance tree).

According to PHYLIP-predicted distances, bacteriophytochrome sequences sharing the same effector, but occurring in different species, have closer relationships than bacteriophytochrome sequences linked to different effectors within a single species. For the majority of effectors, this is corroborated by sequence identities of PAS-GAF-PHY core modules. Based on this observation, we conclude that the occurrence of multiple phytochrome-linked effector combinations is predominantly based on horizontal gene transfer, a well-established major player in microbial evolution [29], rather than recurring internal recombination events. This is further supported by the fact that relatively closely related sequences also occur in different phyla and classes of the bacterial kingdom. Nevertheless, there is also no strict clustering according to effector domains, suggesting that comparable sensor–effector recombination events have happened multiple times, with different fusions improving species fitness.

For the selected organisms containing GGDEF or GGDEF-EAL outputs along other bacteriophytochrome-linked effectors, frequently a closer relationship is observed between identical sensor–effector combinations rather than the bacteriophytochrome sequences within the respective organism. While this shows that functional systems are present in many organisms, it also indicates that successful recombination events which lead to novel red light-regulated systems have been rather rare.

### A defined set of linker lengths is found in naturally occurring PadCs

In this study, we focussed on a group of bacteriophytochromes for which the linker connecting sensor and effector domain has emerged as an important structural and functional element [5–8], i.e. the family of phytochrome-activated diguanylate cyclases. PadCs are defined by the presence of a set of essential motifs and residues: A highly conserved cysteine in the NTS, which covalently binds to the chromophore [30, 31] (C17 in *Is*PadC), the DIP motif in the GAF domain cofactor binding pocket [32], the PRXSF motif in the PHY tongue [10] and the DGC activity defining GGDEF/GGEEF [33] motif. PadC-EALs are required to additionally feature the EAL [15] or EXL motif [34], which is part of the EAL-PDE active site [35], where X can be any hydrophobic, aliphatic residue. The number of sequences obtained when searching the NCBI protein database [36] using the iterative PSI-blast algorithm [37] under these constraints ranged around 200 for PadCs (~ 140) and PadC-EALs (~ 60) combined (consult Supp. Tables 1–5 for additional information).

**Fig. 3:**
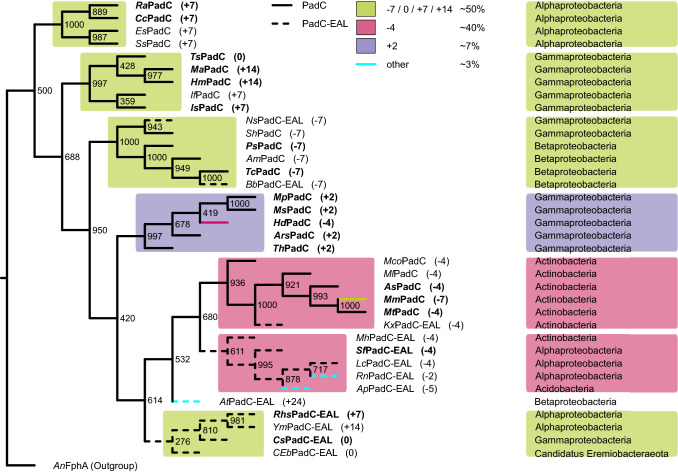
Bootstrap tree of representative PadC (continuous) and PadC-EAL (dashed) homologs, relative linker length prevalence is indicated for PadCs and PadC-EALs combined. Bold entries represent biochemically characterised homologs. PadCs cluster according to linker length and linker lengths differing by multiples of seven are represented in green, − 4 in pink and + 2 in purple; all others are highlighted in turquoise. The corresponding taxonomy class is provided on the right for every homolog

In order to further investigate the evolutionary relationship amongst PadC sequences, we subjected a structure-oriented global alignment (complete alignment in Supp. Figure 1), of which a subset was previously used to motivate the characterisation of *Mp*PadC [8], to a PHYLIP distance analysis with 1000 delete-half Jackknife sub-alignments [38]. The resulting phylogenetic tree (Fig. 3, see Supp. Tables 1 and 2 for NCBI accession numbers and additional information) constructed from representative PadC and PadC-EAL sequences uses the BphP-like fungal phytochrome FphA from *Aspergillus nidulans* [39, 40] as an outgroup. This bootstrap tree clearly highlights the importance of the coiled-coil linker element and we therefore include the corresponding linker length information in all homolog descriptions as superscript from now on—e.g.* Is*PadC^+7^, corresponding to a linker length of + 7 residues relative to the reference system *Ts*PadC^0^ (*Thioalkalivibrio* sp. ALMg3, with a 28 residues long linker). The latter is defined as the number of amino acid residues between a conserved hydrophobic residue in the terminal PHY-helix (I495 in *Is*PadC^+7^) and the DXLT motif at the beginning of the effector domain. In our phylogenetic analysis, three homologs were included despite not corresponding to the definition of PadCs and PadC-EALs as described above—*Hm*PadC^+14^ (*Halomonas meridiana*), a homolog missing all essential motifs and residues that regardlessly clusters tightly with *Is*PadC^+7^, as well as *Bb*PadC-EAL^−7^ (*Betaproteobacteria bacterium* SCN2) and *Ns*PadC-EAL^−7^ (*Nevskia soli*), which feature an EAL motif mutated to EAF, most likely resulting in no EAL-PDE activity in those systems.

**Fig. 4:**
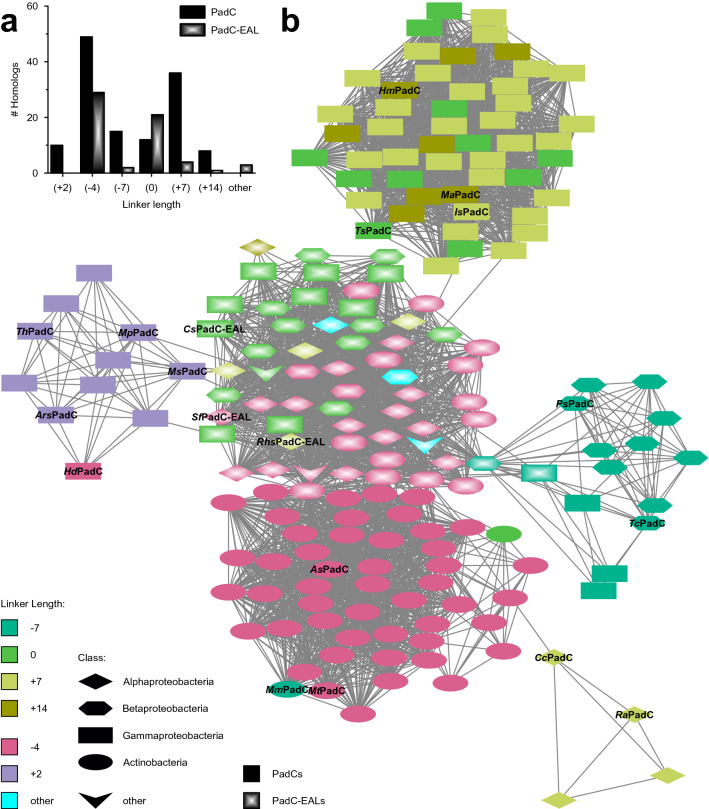
Evolutionary distribution of various PadC linker lengths.** a** Relative frequencies of naturally occurring linker lengths in PadCs (black) and PadC-EALs (opaque). **b** Sequence similarity network of PadCs and PadC-EALs. Nodes correspond to different homologs; edges represent BLAST-generated bitscores (S) above a minimal threshold of 380. Linker lengths divisible by seven are shown in shades of green, PadCs^+2^ in purple and PadCs^−4^ as well as PadC-EALs^−4^ in pink; turquoise denotes other rare linker lengths. The different node shapes refer to the phylogenetic class. All expressed and/or biochemically characterised homologs are indicated by name as referred to in previous publications [5, 7, 8] or this manuscript

**Fig. 5:**
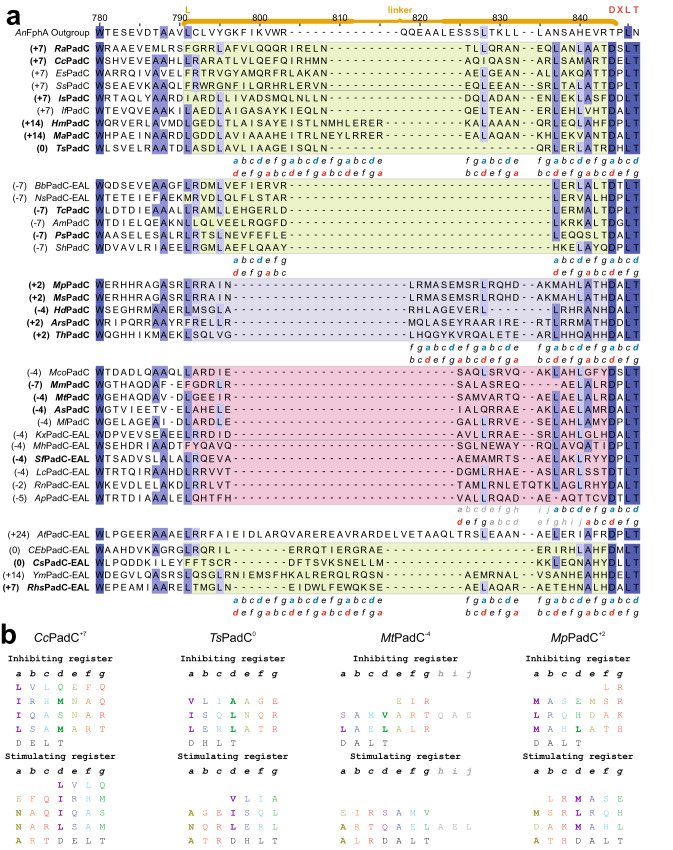
Sequences and register predictions of the coiled-coil linker elements.** a** Alignment of the coiled-coil linker, with linker lengths represented by colour (green = divisible by seven, pink = − 4, purple =  + 2). Highlighting of residues corresponds to the degree of conservation (hues of dark blue to the respective background colour =  ≥ 84%, ≥ 68% ≥ 40%, < 40%). Heptad repeat patterns typical for coiled-coils are listed for inhibiting (blue) and stimulating (red) registers, grey residues correspond to the overwinding postulated for PadCs^−4^ [43]. Bold entries represent investigated homologs. **b** Heptad repeat definitions in both inhibiting and stimulating registers for *Cc*PadC^+7^, *Ts*PadC^0^, *Mt*PadC^−4^ and *Mp*PadC^+2^; see [5] for a representation of *Is*PadC^+7^. Coiled-coil stabilising residues (hydrophobics and Asn) are bold

Interestingly, while only a limited set of linker lengths is found within the compiled set of PadCs, the group of PadC-EALs additionally features the unique linker lengths of − 5, − 2 and + 24 (*Ap*PadC-EAL^−5^, *Acidisarcina polymorpha*; *Rn*PadC-EAL^−2^, *Roseomonas nepalensis*; *At*PadC-EAL^+24^, *Aquabacterium tepidiphilum*). It should also be noted that no single organism features more than one PadC homolog with different linker lengths. Figure 4 depicts the complete set of PadCs in the form of a sequence similarity network (SSN), with BLAST-generated [41, 42] bitscores (S) above a threshold of 380 serving as edges and nodes representing homologs coloured according to linker length. The majority of PadCs (54%) feature linker length differences divisible by seven, which is indicative of the heptad repeat patterns necessary for coiled-coil formation, as has also been confirmed experimentally [5, 6]. Interestingly, with 38% the number of identified homologs with a seemingly non-conforming linker length of − 4 (PadCs^−4^) is surprisingly high and, in addition, a small number of PadCs was found to have a linker length of + 2 (PadCs^+2^) [8]. The relative abundance of all linker lengths is represented in Fig. 4a; and Fig. 5 depicts close-ups of all linker area sequences as well as the residues involved in stabilising the predicted inhibiting and stimulating registers. Whereas PadCs cluster according to linker length in the SSN, at the chosen bitscore cut-off, all PadC-EALs group together regardless of linker length. At increasing cut-offs, PadC-EALs also separate into linker length-specific clusters as seen in the bootstrap tree; however, individual PadC subfamilies are no longer linked to the SSN at that point.

### Different linker length families are most likely based on parallel evolution

The phylogenetic tree (Fig. 3) features strong clustering according to linker length, with one major branch of mixed linker lengths divisible by seven including both *Is*PadC^+7^ and *Ts*PadC^0^ [5], as corroborated by bootstrap values. High bootstrap values for each individual branch indicate that they are significant as separate branches (> 950, i.e. a 95% confidence interval), whereas the relatively low bootstrap values defining relation of the branches to each other point most likely towards parallel evolution of individual linker length families. The exception to this trend of parallel evolution is the extensive cluster of PadCs^−4^ and PadC-EALs^−4^, which are closely related to each other, though not significant as an individual branch. *Kx*PadC-EAL^−4^ (*Kineococcus xinjiangensis*), interestingly, clusters with PadCs^−4^ rather than PadC-EALs^−4^, as confirmed by a bootstrap value of 1000. All PadCs^−4^, as well as roughly 50% of the PadC-EAL^−4^ homologs, belong to the class of Actinobacteria (as visualised in the SSN depicted in Fig. 4), indicating that PadCs with a linker length of − 4 evolved from PadC-EALs^−4^ through loss of the EAL domain. The odd PadC^−4^ family member *Mm*PadC^−7^ (*Microbacterium mangrove*—see Fig. 3 and Supp. Fig. 2) is present in an Actinobacterium, like its PadC^−4^ branch members. In contrast, *Hd*PadC^−4^ (*Halospina denitrificans*), which clusters in the PadC^+2^ group, is found in a Gammaproteobacterium, further reinforcing the PHYLIP-predicted relationships of both homologs in spite of their divergent linker lengths. Annotated linker lengths of both homologs were confirmed as correct through re-sequencing of DSMZ DNA. That the few observed outliers also feature linker lengths observed in other branches further shows the high evolutionary pressure restricting linkers to a confined length and to a lesser degree sequence space.

In general, PadC-EALs—though not equally robust in their linker length-related clustering as PadCs—firmly group together. Moreover, in a bootstrap tree of the EAL domains only (Supp. Figure 3), no individual branches are statistically significant, suggesting a close evolutionary relationship of the EAL domains. The strong conservation of length observed for the linker element between GGDEF and EAL domains in addition to numerous conserved residues (Supp. Fig. 4) further supports the hypothesis of a common GGDEF-EAL ancestor. This conservation of the EAL-linker is seen even in the two exceptions to the cluster of PadC-EALs. *Bb*PadC-EAL^−7^ and *Ns*PadC-EAL^−7^, both featuring a linker length of – 7, are more closely related to PadCs^−7^ than to other PadC-EAL homologs. Interestingly, when considering relationships of the EAL domains, PadC-EALs^−7^ are not significant as a branch despite the loss of residues necessary for catalytic competence.

### Homologs with a functional coiled-coil linker permit efficient red light-induced regulation of enzymatic activity

As previously published [8] and outlined in the introduction, two PadCs of different linker lengths that were characterised in greater detail—*Is*PadC^+7^ and *Mp*PadC^+2^—distinctly differ in their biochemical behaviour. In order to examine how far these observed disparities in light-controlled regulation of enzymatic activity, as well as spectral behaviour, correlate with linker length, we have biochemically characterised additional representative PadCs of each branch. The different homologs were selected based on phylogenetic diversity in an attempt to address how specific characteristics are preserved within branches and linker length groups. In addition to archetypic representatives of the individual groups, *Hd*PadC^−4^ and *Mm*PadC^−7^ were investigated, to see whether their behaviour is influenced more strongly by linker length or overall sequence similarity. Three representatives of the PadC-EAL subgroup were included as well—*Sf*PadC-EAL^−4^ (*Salinihabitans flavidus*), *Rhs*PadC-EAL^+7^ (*Rhodobacter* sp. JA431) and *Cs*PadC-EAL^0^ (*Catenovulum sediminis*). The three latter homologs showed different degrees of oligomerisation and degradation, are regulated by red light and all three feature DGC activity. *Rhs*PadC-EAL^+7^ is the only homolog investigated for which EAL-PDE activity was measured as well, and its partial cleavage pattern corresponds to what has previously been reported for the *Rhodobacter sphaeroides* PadC-EAL homolog [14], a member of the PadC-EAL^−4^ family. Due to low overall yields and the observed mixtures of oligomeric species of full-length and partially truncated forms of the proteins, a quantitative description of kinetic and spectral properties would go beyond the scope of this manuscript.

#### Homologs with linker lengths divisible by 7

From the three branches containing linker lengths divisible by seven, a total of six homologs were characterised in detail (see Supp. Tables 1 and 2 for NCBI accession numbers and taxonomy). *Cc*PadC^+7^ (*Cereibacter changlensis*) and *Ra*PadC^+7^ (*Rhodoligotrophos appendicifer*) represent a branch exclusively comprised of homologs with a linker length of + 7. Of the PadC^−7^ branch, only *Ps*PadC^−7^ (*Pusillimonas maritima*) could be characterised, as expression levels of *Tc*PadC^−7^ (*Thauera chlorobenzoica*) were too low to allow efficient purification. The same held true for *Hm*PadC^+14^, the homolog lacking all essential motifs and residues. From the central branch of homologs with a canonical linker length, *Ma*PadC^+14^ (*Marinimicrobium agarilyticum*), featuring a linker length of + 14, was previously characterised in addition to *Is*PadC^+7^ and *Ts*PadC^0^ [5].

**Fig. 6:**
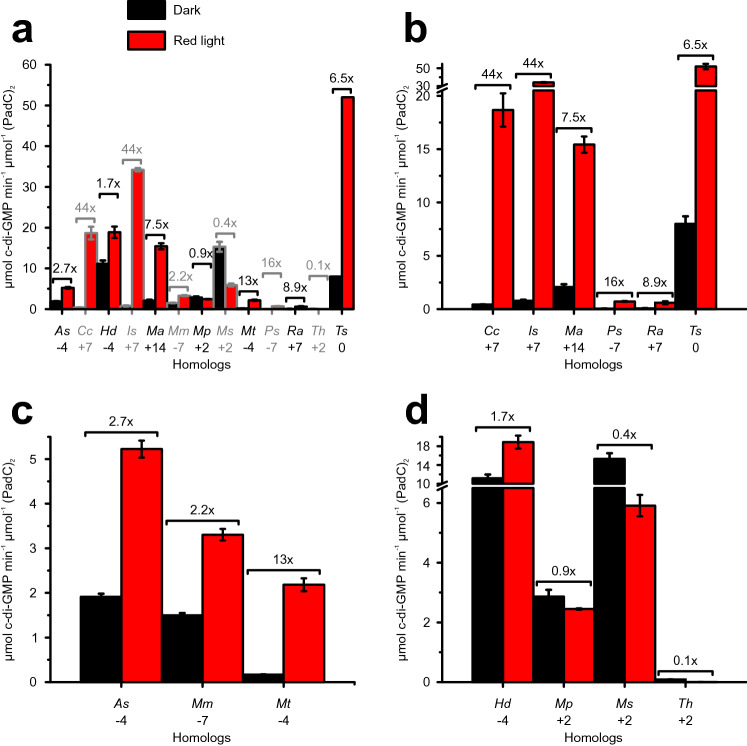
Specific DGC activities for representative PadCs. All constructs are compared at 200 µM GTP and normalised to dimer concentrations of protein, *Is*PadC^+7^, *Ma*PadC^+14^ and *Ts*PadC^0^ specific activities correspond to those previously published [5, 7]. Turnover rates, measured under non-actinic (black) and red light (red) conditions, were calculated from four timepoints each. Error bars represent the SE of the estimate from the linear regression. Fold differences between dark and red light measurements are indicated by brackets above the bars, numbers < 1 denote downregulation of DGC activity. **a** Overall comparison of specific activities measured for every investigated homolog. **b**–**d** Specific activities of PadCs with linker lengths divisible by seven, PadCs^−4^ (including *Mm*PadC^−7^) and PadCs^+2^ (including *Hd*PadC^−4^), respectively

All six homologs listed above show strong red light-dependent upregulation of enzymatic activity, as visualised in Fig. 6a, b and detailed in Supp. Table 6. Dynamic ranges reach from 6.5-fold upregulation, as measured for *Ts*PadC^0^, to a roughly 40-fold increase seen in *Is*PadC^+7^ or *Cc*PadC^+7^. Neither are these differences in light regulation homogenous within individual branches of the phylogenetic tree, nor are absolute turnover rates under both red light and non-actinic conditions. However, a significant increase in enzymatic activity upon illumination is observed for all investigated PadC homologs with a linker length divisible by seven.

**Fig. 7:**
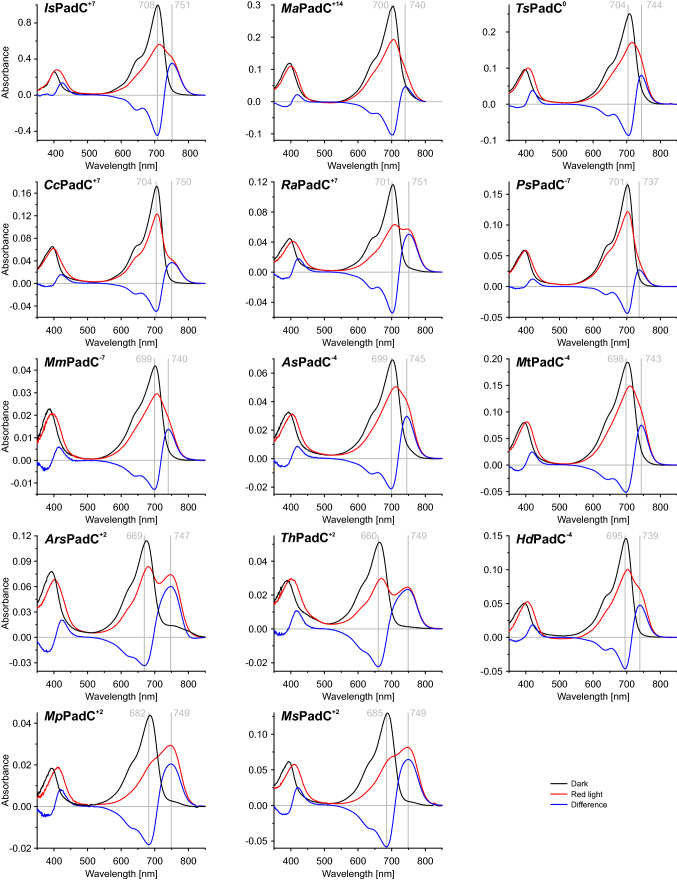
UV/Vis absorption spectra of all characterised homologs. Dark state (black), red light (red) and difference (light—dark; blue) in-solution spectra obtained at 20 °C and with identical illumination protocols

Similarly, all homologs of this group feature spectra with a non-canonical light state (see Fig. 7), as previously published for *Is*PadC^+7^ [5, 6]. Interestingly, the relative contributions of P_fr_ and P_r_-resembling [44] Q-bands to the light-activated spectra differ substantially amongst homologs, indicating different contributions of the canonical P_fr_ state to the individual light state spectra [8]. Wavelengths of both P_r_ and P_fr_ Q-band maxima are relatively similar amongst PadCs with linker lengths divisible by seven; with P_r_ maxima ranging from 700 to 708 nm and P_fr_ Q-band maxima from 737 to 751 nm. Within this group, P_fr_ state stabilities vary considerably, with mean lifetimes averaging from 1 to 2 min (*Cc*PadC^+7^, *Is*PadC^+7^ [5]) to approximately 1 h (*Ma*PadC^+14^, *Ts*PadC^0^ [5]).

#### Homologs belonging to the branch featuring predominantly linker lengths of − 4

P_r_ and P_fr_ Q-band absorption maxima of PadCs^−4^—*As*PadC^−4^ (*Agromyces* sp. Leaf222) and *Mt*PadC^−4^ (*Microbacterium trichothecenolyticum*)—as well as the closely related *Mm*PadC^−7^ are found to be in a similar range; 698 / 699 nm and between 740 and 745 nm, respectively. Interestingly, the extent of P_fr_ contributions to the steady-state spectrum during red light illumination (680 nm) in this group is more homogenous than within the branches with linker lengths divisible by 7; and all three homologs feature non-canonical light state spectra. Mean P_fr_ state life times were measured to be in the range of one hour.

The dynamic ranges of light activation measured for this group are generally noticeably smaller (ranging from 2.2 to 13 fold upregulation) than observed for linker families with complete heptad repeats, but specific turnover numbers are considerably more uniform (see Fig. 6 and Supp. Table 6). *Mm*PadC^−7^ shows behaviour very similar to that of *As*PadC^−4^; red light illumination triggers upregulation of enzymatic activity in both homologs, but on a smaller scale than observed for *Mt*PadC^−4^ or PadCs belonging to the sub-families with linker lengths divisible by seven.

#### Homologs belonging to the branch featuring predominantly linker lengths of + 2

In contrast, homologs with a linker length of + 2 differ substantially in their behaviour from all other PadCs, as recently described in detail for *Mp*PadC^+2^ [8]. In addition to this representative, four additional homologs from this branch of the phylogenetic tree were characterised—*Ars*PadC^+2^ (*Arhodomonas* sp. KWT), *Ms*PadC^+2^ (*Marinobacter* sp. T13-3), *Th*PadC^+2^ (*Thiohalospira halophila*) as well as *Hd*PadC^−4^. Similar to members of the PadC^−4^ branch, *Hd*PadC^−4^ shows some upregulation of enzymatic activity upon illumination (a factor of 1.7). *Mp*PadC^+2^, *Ms*PadC^+2^ and *Th*PadC^+2^, in contrast, feature either almost no effect of illumination (*Mp*PadC^+2^) or even red light-induced downregulation of enzymatic turnover (*Ms*PadC^+2^, *Th*PadC^+2^) up to only a tenth of the activity measured under non-actinic conditions (Fig. 6, Supp. Table 6). Contrary to PadCs with a linker length divisible by seven, which feature highly efficient inhibition in the absence of red light, a relatively high basal activity was observed for *Mp*PadC^+2^ and *Ms*PadC^+2^ as well as *Hd*PadC^−4^. Interestingly, the HPLC-based assay used to quantify enzymatic activity also exposed a secondary product peak—considerably smaller than that measured for c-di-GMP—in *Th*PadC^+2^. *Ars*PadC^+2^, to our surprise, produced only this additional, as-of-yet unidentified compound, and no measurable quantities of c-di-GMP.

In-solution spectra further emphasise the differences between the PadC^+2^ family and homologs with other linker lengths. While P_fr_ Q-band absorption maxima fall within the scope observed for the previously introduced groups (ranging from 739 to 750 nm), P_r_ Q-maxima show considerable variations (660–695 nm). The most extreme value was measured for *Th*PadC^+2^. Only one additionally investigated member of the PadC^+2^ family, *Ms*PadC^+2^, features a symmetric, canonical light state as has been reported for *Mp*PadC^+2^ [8]; in contrast, light state spectra measured for *Ars*PadC^+2^, *Th*PadC^+2^ and *Hd*PadC^−4^ rather feature non-canonical spectra as observed for *Is*PadC^+7^, albeit to a lesser degree. P_r_ state recovery kinetics feature mean lifetimes of approximately one hour for *Hd*PadC^−4^, and range up to about 30 h measured for *Mp*PadC^+2^ [8], showing considerably higher P_fr_ state stability than all other linker length families.

### The central helical spine strongly influences effector domain positioning

The ability of a PadC dimer to form a functional coiled-coil in its sensor–effector linker has a profound effect on protein structure as well as dynamics. In the absence of experimentally determined structures, we calculated AlphaFold2 [45] predictions for all representative PadC homologs (see Supp. Figure 5 and 6). 5LLW, the published dark state structure of *Is*PadC^+7^ [5], was part of the array used to train the AlphaFold2 AI [45], eliminating a potential benchmark. According to pLDDT and PAE scores (see Supp. Figs. 7 and 8, respectively), confidence in predictions of the PAS-GAF core and its dimer interface is high. PHY dimerisation and coiled-coil linker architecture are expectedly less confident, and the prediction of the relative GGDEF effector module orientations is not well defined. The latter was expected due to the high degree of freedom observed for the DGC effector in SAXS measurements of *Is*PadC^+7^ [5]. Eventually, the extent of this conformational freedom depends on the degrees of freedom in the PHY domain dimer interface as well as its compatibility with a coiled-coil linker architecture. AlphaFold2 predictions, while not as robust as an experimentally determined structure, still serve to illustrate the intrusive effect of very short linkers (*Ps*PadC^−7^, Supp. Fig. 5b), or the lack of functional coiled-coils (Supp. Fig. 5c). Strong conservation of coiled-coil contacts in the sensor–effector linker reaching all the way to the characteristic wide turn corresponding to the DXLT motif in the DGC effector domain confirmed by coevolution analysis of *Is*PadC^+7^ using the GREMLIN webserver (Supp. Fig. 9) further highlights the importance of the central helical spine in PadC signal integration. Several residues at the C-terminal tip of the GGDEF α_0_ helix and in the GTP-binding site also coevolved with coiled-coil stabilising residues in the linker, highlighting not only direct contacts but also the allosteric regulatory networks at play. Interestingly, no coevolution is observed for the PHY domain dimer interface according to GREMLIN. This is in line with the low degree of conservation of residues in the PHY domain (excluding the PHY tongue) and most likely also reflects the functional divergence of the PHY domain as integrator unit in the different subfamilies of PadCs.

## Discussion

In PadCs specifically [6, 8], and bacteriophytochromes in general [4, 46], the architecture of the central helical spine at the dimer interface greatly affects the degrees of freedom in effector domain orientation. Interestingly, the relatively loose dimer interface suggested by AlphaFold2 predictions of *Mp*PadC^+2^, *Ms*PadC^+2^ and *Th*PadC^+2^ structures correlates with the comparatively open PHY interface conformation observed for the *Deinococcus radiodurans* phytochrome [27, 47, 48]. In the absence of a functional coiled-coil, due to the incomplete heptad repeats of a + 2 linker, the central helical spine underlies fewer constraints and can be considerably more dynamic than observed for *Is*PadC^+7^ and its linker length family members. Additionally, it has been observed that even a functional linker length does not suffice to override the effect of the *Mp*PadC^+2^ PHY dimer interface [8]. In the context of the well-established light-induced structural rearrangements of the PHY tongue [10, 27], this could imply different signal integration mechanisms depending on relative PHY dimer interactions.

Even though PHY domain dimerisation was shown to be a key player in signal integration of PadCs [8], the PHY domain itself—excluding the tongue region—is subject to relatively little evolutionary pressure according to the low degree of sequence conservation (Supp. Fig. 1). Based on its importance for PadC functionality we conclude that the PHY dimer interface provides the evolutionary playground for adapting to various effectors. Whereas the PAS-GAF core is rigidly conserved, orientation of the PHY dimer interface and the central helical spine remains malleable. Potentially, this could also be one explanation for why PadCs tend to allow higher dynamic ranges than many PHY-less cyanobacteriochrome-based diguanylate cyclases [49]. However, tight interaction of the central helical spine is not necessarily a functional prerequisite for other bacteriophytochromes, as observed for the phytochrome-linked histidine kinase/phosphatase of *Deinococcus radiodurans* with its relatively loose PHY domain interaction [50] or the *Xanthomonas campestris* BphP where breaks of the helical spine affect the quaternary assembly [46]. Nonetheless, neither regulation mechanism appears applicable to PadCs and the high degree of conformational flexibility observed for their GGDEF domains [5]. Modulation of DGC activity is known to be regulated by either dimerisation events or reorganisation of input domains enabling productive encounter [33].

Still, light-controlled regulation of enzymatic activity in PadCs depends strongly on PHY dimer interface interactions and coiled-coil register switching [5, 8]. Interestingly, the importance of register switching in coiled-coils influencing DGC activity has recently also been shown for a Rec-GGDEF system [51]. In PadCs, a balance between stabilisation of inhibiting and stimulating registers appears to provide the biophysical framework for efficient register switching, as observed for *Is*PadC^+7^ or *Cc*PadC^+7^ (Fig. 5b). In *Is*PadC^+7^, variants with additional hydrophobic residues in *a* or *d* position of the inhibiting or stimulating register, respectively, suffice to trap the protein in either an inhibited or constitutively active conformation, respectively [6]. However, as seen in the linker regions of the PadC^+2^ family (Fig. 5), a loss of the heptad repeat pattern as well as low conservation of hydrophobic residues in *a* and *d* positions impede the efficient stabilisation of different coiled-coil arrangements, thus implying a different mechanism for the light inhibition of DGC activity observed for some of its family members. For PadCs^−4^, on the other hand, the interference with coiled-coil formation is less pronounced than for PadCs^+2^, as the loss of four residues corresponds to a deletion of roughly one helical turn. In this case, the − 4 deletion is compensated by overwinding of the coiled-coil resulting in a 10 amino acid stretch for 3 turns as shown in Fig. 5 [43, 52]. In analogy to the inhibiting and stimulating registers observed for the families with classical heptad repeats, similar alternative register architectures can be identified in the PadC^−4^ subfamily (Fig. 5b). In this respect, it is not surprising that the biochemical behaviour of this linker length family more closely resembles that of the branch including *Is*PadC^+7^, albeit on average with lower dynamic ranges of enzymatic light activation and in line with the roughly fourfold upregulation observed for the *R. sphaeroides* BphG1 EAL-less construct [14].

The evolutionary behaviour of bacteriophytochromes in general complements what is observed on a smaller scale for linker length constraints in PadCs. Across the range of naturally occurring bacteriophytochromes, more than one functionality can be linked to a biliverdin-based sensory module even within individual organisms, as well-established for Agp1 and Agp2 in *Agrobacterium tumefaciens* [53] or the multiple BphPs identified in *Rhodopseudomonas palustris* [54], and representatives tend to cluster in distance trees according to their output domain. Within an effector family, amino acid substitutions with varying functional consequences can happen occasionally, however, the resulting sequences are only retained if they either improve overall fitness, or generate no additional metabolic burden. We believe that of such random deletions or insertions in PadC linkers, only few survived, which were the origins of *Mm*PadC^−7^ in the PadC^−4^ branch and *Hd*PadC^−4^ in the + 2 branch. The considerable evolutionary pressure on PadC linker length is further corroborated by the very distinct groups of linker lengths that are observed. With the exception of PadC-EALs, no naturally occurring linker lengths other than − 4 and + 2 consisting of incomplete heptad repeats were identified. A similar conservation of linker length is seen in LOV-regulated effector domains and especially pronounced for LOV-regulated DGCs [55]. We have previously suggested that PadCs with a linker length of + 2 might not function as light-sensing signalling systems based on the comparison of apo- to holoprotein DGC activity as well as P_fr_ state stability, thus the loss in linker length may rather be a symptom of a successful functional adaptation than the cause [8]. A functional importance of apoforms has also been shown for other bacteriophytochromes in low light responses [56]. The unidentified enzymatic turnover of a side-product observed for *Ars*PadC^+2^ as well as—to a lesser extent—*Th*PadC^+2^ further suggests that this particular linker family serves a different function on a cellular signalling level than other PadCs, negating the evolutionary pressure on coiled-coil linker architecture. From an evolutionary perspective, protein function can change for particular homologs of a family as they are recruited for a different purpose without requiring extensive alterations to the sequence [57]. Moreover, Mantoni et al. [58] have recently shown that if productive active site formation at the GGDEF dimer is prevented, these domains are capable of slowly converting GTP to GMP. HPLC–MS analysis of the unknown product produced by *Ars*PadC^+2^ as well as *Th*PadC^+2^ does, however, not correspond to GMP, nor to GDP, 5’-pGpG or pppGpG (the intermediate frequently observed for DGC reactions [59]). Basal DGC activity levels measured for *Th*PadC^+2^ are notably low and non-existent in *Ars*PadC^+2^, suggesting that in some PadCs with a non-prototypical linker length the productive encounter of the effector domains is highly inefficient, allowing for production of various side-products.

The hypothesis of parallel development of different PadC linker length families based on low bootstrap values between individual branches (Fig. 3) is further supported by the relatively strong conservation of taxonomic classes, as also visualised in the SSN (Fig. 4). Within the family of PadCs, many significant linker length branches correspond exclusively to Alpha- (PadCs^+7^) or Gammaproteobacteria (PadCs^+2^, branch containing *Is*PadC^+7^); PadCs^−7^ contain both Alpha- and Betaproteobacteria, whereas the branch of PadCs^−4^ contains Actinobacteria only. In contrast, PadC-EALs feature Acidobacteria as well as Alpha-, Beta-, Gammaproteobacteria and Actinobacteria. However, phylogenetic clustering by linker length has been shown to be less robust in PadC-EALs, as corroborated by the bootstrap values. PadC-EALs appear to be more flexible overall when it comes to linker length, featuring three unique linker lengths of − 5, − 2 and + 24, which could be an indicator that their EAL-PDE activity is of higher relevance to cellular signalling than that of the DGC output module.

On an evolutionary level, we believe that fusion events of GGDEF-EAL couples to bacteriophytochrome cores were rare during evolution and that the EAL domain was then repeatedly lost, yielding the groups of PadCs^−4^ and PadCs^−7^. This conclusion is based solidly on the relationship of PadC-EAL^−7^ EAL domains to other homologs’ as well as the degree of conservation observed for the EAL-linker length and the numerous conserved residues between DGC and EAL domains (see Supp. Fig. 4), which is highly unlikely to have developed repeatedly and independently by coincidence. Moreover, the structural element preceding the EAL domain has been demonstrated to be a structurally well-defined helical linker in GGDEF-EAL couples [60] and also an important EAL-regulatory element in BLUF-EAL systems [61].

*Ns*PadC-EAL^−7^ and *Bb*PadC-EAL^−7^ further attract attention by clustering with the significant branch of PadCs^−7^. Despite the loss of a highly conserved NTS motif, biliverdin binding and light regulation of DGC activity do not appear to be impeded in this group. In contrast, the EAL motif itself features disruptive mutations to EAF, in addition to mutations in many motifs listed as essential for diguanylate phosphodiesterase activity (e.g. the DDFGTG motif) [34], and we expect neither homolog to feature any EAL-PDE activity. In fact, it has been shown that in proteins featuring a tandem GGDEF-EAL effector module, often one of the two output domains is inactive [22, 34]; however, the catalytically incompetent domain frequently still affects enzymatic activity of the functional output module. Since EAL domains usually require dimerisation for efficient catalysis [34, 62], oligomerisation events of the secondary output domain might influence the conformational sampling of the GGDEF domains in PadC-EALs^−7^.

The majority of phylogenetic relationships observed for PHYLIP-generated distance trees of PadCs is also reflected in sequence similarity-based networks, including the assignment of atypical linker lengths within a branch (*Mm*PadC^−7^, *Hd*PadC^−4^). Use of bitscores (S) rather than E-values means query length does not affect the output value, which allows for comparison of PadCs and PadC-EALs in the same network. Relationships visualised in SSNs have been shown to correlate well with known functional relationships [63], and we confirm that the linker length representation in a sequence similarity network of PadCs and PadC-EALs largely concurs with phylogenetic as well as biochemical observations. Sequence similarity may not permit unambiguous statements about structure or function, however, the visualised relationships detailed in this paper correlate well with known functional relationships between PadC linker length and enzymatic behaviour. Here, we demonstrated that biochemical behaviour of PadCs cannot be transferred par for par to phylogenetic relationships; however, a strong correlation—especially with regard to linker length and the presumably coevolved PHY interface—is clearly observable.

In summary, we were able to show that the high level of conservation and strongly biased evolutionary selection observed for the linker length in phytochrome-activated diguanylate cyclases corresponds prominently to functional characteristics. The rare occurrence of linker length variation within PadC subfamilies only to lengths observed in other subfamilies further highlights the importance of the coiled-coil linker conformation for biochemical function. Successful coiled-coil formation and its effect on orientational sampling of the effector domains affects not only efficiency of signal integration but also spectral properties of the photoreceptor and ultimately regulation of the cellular levels of an important bacterial second messenger. This importance of linker length and sequence on signal integration has also been observed in the past for many naturally occurring systems as well as artificial designs based on bacteriophytochromes for the generation of novel optogenetic tools [4, 64]. Purely sequence-based functional annotation of PadCs, however, remains a challenge. Though doubtlessly the most challenging aspect to automate, the importance of biochemical characterisation in understanding sequence–structure–function space cannot be overstated.

## Materials and methods

### Protein preparation, expression and purification

The coding sequences for all homologs (corresponding NCBI accession numbers are listed in Supp. Table 1) were subjected to *Escherichia coli* codon optimisation and synthesised by GeneArt (Life Technologies). The ordered gene strings were cloned into pETM-11 vectors using the NEBuilder^®^ HiFi DNA Assembly Cloning Kit.

Homologs were expressed and purified as described previously [5]. Briefly, *E. coli* BL21 (DE3) cells containing an additional pT7-ho1 helper plasmid coding for heme oxygenase (HO-1) were used to permit biliverdin synthesis for holoprotein expression. After transformation with the respective PadC-(EAL) plasmids, cells were cultivated in LB medium at 37 °C until OD_600_ 0.45, then cooled to 16 °C. 10 mg L^−1^ δ-aminolevulinic acid were supplied, and expression induced with 0.25 mM isopropyl-β-d-thiogalactopyranoside (IPTG) at an OD_600_ of ~ 0.7. Harvested cells were treated with 100 µg mL^−1^ lysozyme and protease inhibitor cocktail (Roche cOmplete ULTRA tablets) before sonication (5 × 5 min, 70%, pulsed mode; Labsonic L, Sartorius). The soluble fraction was separated by ultracentrifugation (206,000 rcf, 45 min).

Using a Ni^2+^-Sepharose matrix (Ni Sepharose 6 Fast Flow, GE Healthcare), soluble proteins were purified by affinity chromatography. Proteins were eluted (lysis buffer with 250 mM imidazole) following a washing step (lysis buffer with 30 mM imidazole). During overnight dialysis at 4 °C in dialysis buffer (50 mM HEPES pH 7.0, 500 mM NaCl, 2 mM MgCl_2_, 20 mM imidazole) His-tags were cleaved with histidine-tagged tobacco etch virus (TEV) protease (~ 1:16 TEV:substrate). The TEV protease as well as the cleaved tag were removed during another step of affinity chromatography, where the proteins in the flowthrough were concentrated (using ultra centrifugal filters—Amicon Ultra-15; Merck Millipore) and subsequently loaded onto a size exclusion chromatography column (HiLoad 16/60 Superdex 200 prep grade; GE Healthcare) equilibrated in storage buffer (10 mM Hepes pH 7.0, 500 mM NaCl, 2 mM MgCl_2_). Fractions were further concentrated and aliquots flash-frozen in liquid nitrogen to be stored at -80 °C.

### Spectroscopic characterisation

UV–Vis absorption spectra of ~ 2 µM samples (diluted in storage buffer) were acquired using a Specord 200 Plus spectrophotometer (Analytic Jena) at 20 °C. Dark-state P_r_ spectra were measured under non-actinic conditions, minimising contact with measuring light. Light state spectra were recorded after 1 min of red light illumination (660 nm, 20.9 µW/mm^2^, Thorlabs).

P_r_ state recovery kinetics were measured for wavelength maxima and minima of light–dark difference spectra obtained after 1 min of red light illumination (Specord 200 Plus spectrophotometer (Analytic Jena) with 10 ms integration time). As measuring light can affect P_r_/P_fr_ steady states, estimated recovery times should not be over-interpreted quantitatively.

### Kinetic analysis

High-performance liquid chromatography (HPLC) was used to monitor turnover of GTP to c-di-GMP as described previously ([8], adapted from [5]). 400 µM GTP were added to purified protein diluted in reaction buffer (10 mM Hepes pH 7.0, 500 mM NaCl, 50 mM MgCl_2_), yielding a final GTP concentration of 200 µM, after a 1 min incubation at 20 °C under non-actinic light or constant red light illumination (660 nm, 20.9 µW/mm^2^, Thorlabs). After incubation at four different time points under non-actinic or constant red light conditions, samples were thermally inactivated by 1 min incubation at ~ 90 °C. Following a centrifugation step, nucleotides were separated by reversed phase HPLC (ProntoSil C18 ace-EPS column, Bischoff) using an aqueous mobile phase (10 mM K_2_HPO_4_ pH 8.0, 5% MeOH, 5 min) under isocratic conditions at 35 °C. All activities were normalised to the dimer concentration of the respective constructs.

### Phylogenetic analysis

Following an iterative PSI-blast search [37], sequences matching the requirements outlined in the results section, paragraph 2.1, were collected from the NCBI website [65] and compiled according to their official status on 26th January 2021 (accession numbers are listed in Supp. Table 1). Alignments were performed using the T-COFFEE Multiple Alignment Sequence Server (version 11.00) [66, 67], including structural information with the dark state structure for *Is*PadC (advanced: *t_coffee -in* = *file.fa -mode* = *expresso -blast* = *LOCAL—pdb* = *5llw.pdb -evaluate_mode* = *t_coffee_slow -output* = *score_html clustalw_aln fasta_aln score_ascii phylip -maxnseq* = *150 -maxlen* = *2500 -case* = *upper -seqnos* = *off -outorder* = *input -run_name* = *result—multi_core* = *4 -pdb_db* = */db/pdb/derived_data_format/blast/latest/pdb_seqres.fa—protein_db* = */db/ncbi/201511/blast/db/nr.fa -quiet* = *stdout*). The alignment was manually curated in Jalview [68] and reduced for the creation of the final tree, which is included in the paper, to four representatives per branch, excluding outliers; with sequences annotated as “hypothetical” excluded. Individual sequences were chosen based on maximum diversity and distance. Distance as well as bootstrap trees of the curated alignment were created using the PHYLIP 3.6 package (SEQBOOT, PROTDIST, FITCH and CONSENSE, [38]). 1000 Jackknife sub-alignments were created using SEQBOOT and subjected to a bootstrapped protein distance analysis. The fungal phytochrome FphA from *Aspergillus nidulans* was used as an outgroup and trees were visualised using Figtree [69]. After re-evaluation of the compiled sequences on 15th November 2021, the entries listed in Supp. Table 5 were removed from the complete collection of PadCs, since they had been re-annotated as obsolete in the meantime (*i.e.* “*This protein record was suppressed because it is no longer annotated on any genome*”). For phylogenetic work as well as sequence similarity networks, the sequence of WP_114419467 (*Ps*PadC) was substituted with the current version WP_119515934; however, the sequence of the biochemically characterised construct correlates with the obsolete entry. KQM84620 (*As*PadC), now listed as “*hypothetical*”, was left in the alignment as no surrogate of the same length was found.

For comparison of bacteriophytochromes with different effectors, InterPro was searched for entries with a PAS (IPR013654)—GAF (IPR003018)—PHY (IPR013515) architecture. From all domain structures with ≥ 5 hits on 7^th^ April 2021, those with organisms present in more than one architecture family were chosen. A list of representative sequences as depicted in Fig. 2 can be found in Supp. Table 6; alignments were generated with standard T-COFFEE settings and distance trees generated using the PHYLIP package.

### Sequence similarity networks

From the complete set of PadC and PadC-EAL sequences compiled as described above (see Supp. Table 3), databases were created using the *makeblastdb* option of NCBI BLAST + [65], followed by an all-vs-all BLAST. Networks visualised in Cytoscape 3.8.2 [70] represent BLAST-generated bitscores (S) as edges, which were restricted to values higher than 380. Of a given pair of bitscores (S) for two sequences, only the higher one was kept, as described in [63]

### AlphaFold2

Structure predictions were computed using AlphaFold v2.1.0 [45] in a Jupyter notebook as provided by [71]; with default settings, and sequences and dimeric configuration provided as input. The results with the best overall PAE score (Supp. Fig. 8) are presented in this paper, plDDT values for which are depicted in Supp. Fig. 7.

### Analysis of coevolution

Coevolution of PadCs was addressed using the Gremlin server of the Baker lab (http://gremlin.bakerlab.org/) [72]. An alignment of all ~ 200 PadC and PadC-EAL sequences (provided in Supp. Table 3) featuring the PSM-GGDEF parts and *Is*PadC as reference was not sufficient for identification of critical coevolving residues. Therefore, the alignment was extended by the HHblits algorithm using 8 iterations and an *E*-value cut-off of 10^–4^. While still somewhat noisy, the coevolution pattern clearly supports the functional role of previously known phytochrome and GGDEF elements.

## Supplementary Information

Below is the link to the electronic supplementary material.

Supplementary file1 (PDF 6169 KB)
