# Supplementary material for: Characterisation of sequence–structure–function space in sensor–effector integrators of phytochrome-regulated diguanylate cyclases
Source: Photochem Photobiol Sci. 2022 Jul 5;21(10):1761–79. doi: 10.1007/s43630-022-00255-7 (PMC9587094; doi:10.1007/s43630-022-00255-7)
Supplement: Supplementary file 1 — Supplementary file1 (PDF 6169 KB) [file 43630_2022_255_MOESM1_ESM.pdf]

Supplementary Materials for

Characterization of Sequence-Structure-Function Space in Sensor-Effector Integrators of Phytochrome Regulated Diguanylate Cyclases

Böhm Cornelia[1,2], Gourinchas Geoffrey[1,3], Zweytick Sophie[1], Hujdur Elvira[1], Reiter Martina[1], Trstenjak Sara[1], Sensen Christoph Wilhelm[2,4] and Winkler Andreas*[1,2]

Author Information

[1]Institute of Biochemistry, Graz University of Technology, 8010 Graz, Austria

[2]BioTechMed-Graz, 8010 Graz, Austria

[3]Department of Integrated Structural Biology, Institut de Génétique et de Biologie Moléculaire et Cellulaire (IGBMC), 67404 Illkirch, France

[4]Hungarian Centre of Excellence for Molecular Medicine, Római körút 21, 6723 Szeged, Hungary

*Corresponding author: Andreas.Winkler@TUGraz.at; +43 316 873 - 6457

This file includes:

- Supplementary Tables 1 – 6
- Supplementary Figures 1 – 8

Supp. Table 1: Representative PadCs and PadC-EALs as presented in phylogenetic trees *(Fig. 3 and Supp. Fig. 3) – linker length and NCBI protein accession number. DSMZ numbers of HdPadC^{-4} * and MmPadC^{-7} **: 15505 & 28240.*

Homolog	Organism	Length	LL		Accession No.
*An*FphA	*Aspergillus nidulans*	1280			CAI30283
*Am*PadC^{-7}	*Algicoccus marinus*	659	21	-7	WP_108620250
*Ars*PadC^{+2}	*Arhodomonas sp.* KWT	688	30	+2	WP_163139709
*As*PadC^{-4}	*Agromyces sp.* Leaf222	706	24	-4	KQM84620
*Cc*PadC^{+7}	*Cereibacter changlensis*	686	35	+7	WP_170139737
*Es*PadC^{+7}	*Erythrobacter sp.* QSSC1-22B	692	35	+7	WP_067609350
*Hd*PadC^{-4} *	*Halospina denitrificans*	686	24	-4	WP_133735568
*Hm*PadC^{+14}	*Halomonas meridiana*	670	42	+14	WP_159340624
*If*PadC^{+7}	*Idiomarina fontislapidosi*	689	35	+7	WP_110572772
*Is*PadC^{+7}	*Idiomarina sp.* A28L	683	35	+7	WP_007419415
*Ma*PadC^{+14}	*Marinimicrobium agarilyticum*	700	42	+14	WP_027329460
*Mco*PadC^{-4}	*Mycobacterium cookie*	669	24	-4	WP_163780714
*Ml*PadC^{-4}	*Microbacterium lemovicicum*	665	24	-4	AZS35752
*Mm*PadC^{-7} **	*Microbacterium mangrovi*	640	21	-7	WP_052226828
*Mp*PadC^{+2}	*Marinobacter persicus*	683	30	+2	WP_091706258
*Ms*PadC^{+2}	*Marinobacter sp.* T13-3	685	30	+2	KXS53631
*Mt*PadC^{-4}	*Microbacterium trichothecenolyticum*	650	24	-4	WP_052676815
*Ps*PadC^{-7}	*Pusillimonas maritima*	676	21	-7	WP_119515934
*Ra*PadC^{+7}	*Rhodoligotrophos appendicifer*	667	35	+7	WP_205015307
*Sh*PadC^{-7}	*Salinisphaera hydrothermalis* C41B8	669	21	-7	KEZ78150
*Ss*PadC^{+7}	*Sphingomonas sp.*	690	35	+7	MBA2635521
*Tc*PadC^{-7}	*Thauera chlorobenzoica*	670	21	-7	WP_075148475
*Th*PadC^{+2}	*Thiohalospira halophila*	688	30	+2	WP_159432998
*Ts*PadC^{0}	*Thioalkalivibrio sp.* ALMg3	675	28	0	WP_026331574
*Ap*PadC-EAL^{-5}	*Acidisarcina polymorpha*	951	23	-5	WP_114207983
*At*PadC-EAL^{+24}	*Aquabacterium tepidiphilum*	984	52	+24	WP_119157670
*Bb*PadC-EAL^{-7}	*Betaproteobacteria bacterium* SCN2	922	21	-7	KAB2309365
*CEb*PadC-EAL^{0}	*Candidatus Eremiobacteraeota bacterium*	978	28	0	MBD5655562
*Cs*PadC-EAL^{0}	*Catenovulum sediminis*	970	28	0	WP_143870274
*Kx*PadC-EAL^{-4}	*Kineococcus xinjiangensis*	946	24	-4	WP_158257126
*Lc*PadC-EAL^{-4}	*Lichenicola cladoniae*	936	24	-4	WP_171837799
*Mh*PadC-EAL^{-4}	*Mycolicibacterium hodleri*	942	24	-4	WP_142550055
*Ns*PadC-EAL^{-7}	*Nevskia soli*	930	21	-7	WP_180540017
*Rn*PadC-EAL^{-2}	*Roseomonas nepalensis*	966	26	-2	WP_140884289
*Rhs*PadC-EAL^{+7}	*Rhodobacter sp.* JA431	963	35	+7	WP_097082434
*Sf*PadC-EAL^{-4}	*Salinihabitans flavidus*	941	24	-4	SEO67024
*Ym*PadC-EAL^{+14}	*Yoonia maricola*	960	42	+14	WP_100368535

***Supp. Table 2: Representative PadCs and PadC-EALs as presented in phylogenetic trees** (Fig. 3 and Supp. Fig. 3) – order and class.*

Homolog	Organism	Order	Class
*An*FphA	*Aspergillus nidulans*	Eurotiales	Eurotiomycetes
*Am*PadC^{-7}	*Algicoccus marinus*	Burkholderiales	Betaproteobacteria
*Ars*PadC^{+2}	*Arhodomonas sp.* KWT	Chromatiales	Gammaproteobacteria
*As*PadC^{-4}	*Agromyces sp.* Leaf222	Micrococcales	Actinobacteria
*Cc*PadC^{+7}	*Cereibacter changlensis*	Rhodobacterales	Alphaproteobacteria
*Es*PadC^{+7}	*Erythrobacter sp.* QSSC1-22B	Sphingomonadales	Alphaproteobacteria
*Hd*PadC^{-4}	*Halospina denitrificans*	Oceanospirillales	Gammaproteobacteria
*Hm*PadC^{+14}	*Halomonas meridiana*	Oceanospirillales	Gammaproteobacteria
*If*PadC^{+7}	*Idiomarina fontislapidosi*	Alteromonadales	Gammaproteobacteria
*Is*PadC^{+7}	*Idiomarina sp.* A28L	Alteromonadales	Gammaproteobacteria
*Ma*PadC^{+14}	*Marinimicrobium agarilyticum*	Cellvibrionales	Gammaproteobacteria
*Mco*PadC^{-4}	*Mycobacterium cookie*	Corynebacteriales	Actinobacteria
*Ml*PadC^{-4}	*Microbacterium lemovicicum*	Micrococcales	Actinobacteria
*Mm*PadC^{-7}	*Microbacterium mangrovi*	Micrococcales	Actinobacteria
*Mp*PadC^{+2}	*Marinobacter persicus*	Alteromonadales	Gammaproteobacteria
*Ms*PadC^{+2}	*Marinobacter sp.* T13-3	Alteromonadales	Gammaproteobacteria
*Mt*PadC^{-4}	*Microbacterium trichothecenolyticum*	Micrococcales	Actinobacteria
*Ps*PadC^{-7}	*Pusillimonas maritima*	Burkholderiales	Betaproteobacteria
*Ra*PadC^{+7}	*Rhodoligotrophos appendicifer*	Hyphomicrobiales	Alphaproteobacteria
*Sh*PadC^{-7}	*Salinisphaera hydrothermalis* C41B8	Salinisphaerales	Gammaproteobacteria
*Ss*PadC^{+7}	*Sphingomonas sp.*	Sphingomonadales	Alphaproteobacteria
*Tc*PadC^{-7}	*Thauera chlorobenzoica*	Rhodocyclales	Betaproteobacteria
*Th*PadC^{+2}	*Thiohalospira halophila*	Chromatiales	Gammaproteobacteria
*Ts*PadC^{0}	*Thioalkalivibrio sp.* ALMg3	Chromatiales	Gammaproteobacteria
*Ap*PadC-EAL^{-5}	*Acidisarcina polymorpha*	Acidobacteriales	Acidobacteria
*At*PadC-EAL^{+24}	*Aquabacterium tepidiphilum*	Burkholderiales	Betaproteobacteria
*Bb*PadC-EAL^{-7}	*Betaproteobacteria bacterium* SCN2		Betaproteobacteria
*CEb*PadC-EAL^{0}	*Candidatus Eremiobacteraeota bacterium*		
*Cs*PadC-EAL^{0}	*Catenovulum sediminis*	Alteromonadales	Gammaproteobacteria
*Kx*PadC-EAL^{-4}	*Kineococcus xinjiangensis*	Kineosporiales	Actinobacteria
*Lc*PadC-EAL^{-4}	*Lichenicola cladoniae*	Rhodospirillales	Alphaproteobacteria
*Mh*PadC-EAL^{-4}	*Mycolicibacterium hodleri*	Corynebacteriales	Actinobacteria
*Ns*PadC-EAL^{-7}	*Nevskia soli*	Nevskiales	Gammaproteobacteria
*Rn*PadC-EAL^{-2}	*Roseomonas nepalensis*	Rhodospirillales	Alphaproteobacteria
*Rhs*PadC-EAL^{+7}	*Rhodobacter sp.* JA431	Rhodobacterales	Alphaproteobacteria
*Sf*PadC-EAL^{-4}	*Salinihabitans flavidus*	Rhodobacterales	Alphaproteobacteria
*Ym*PadC-EAL^{+14}	*Yoonia maricola*	Rhodobacterales	Alphaproteobacteria

***Supp. Table 3: PadC and PadC-EAL sequences represented in the SSN** (Fig. 4) – NCBI protein accession number, organism, class and linker length (LL); for identification of representative homologs depicted in phylogenetic trees, consult Supp. Table 1*

Accession Number	Organism	Class	LL
	**PadCs**		
WP_157414452	*Agromyces allii*	Actinobacteria	-4
NLY26520	*Alcaligenaceae bacterium*	Betaproteobacteria	-7
WP_011629241	*Alkalilimnicola ehrlichii*	Gammaproteobacteria	7
ABI56846	*Alkalilimnicola ehrlichii* MLHE-1	Gammaproteobacteria	7
WP_092568142	*Aidingimonas halophila*	Gammaproteobacteria	7
WP_126791882	*Aliidiomarina haloalkalitolerans*	Gammaproteobacteria	0
WP_108620250	*Algicoccus marinus*	Betaproteobacteria	-7
WP_111569169	*Aliidiomarina maris*	Gammaproteobacteria	7
WP_189085369	*Agromyces mediolanus*	Actinobacteria	-4
WP_157426742	*Agromyces salentinus*	Actinobacteria	-4
WP_126808506	*Aliidiomarina shirensis*	Gammaproteobacteria	7
WP_104189763	*Arthrobacter sp.* 9E14	Actinobacteria	-4
WP_153684347	*Agromyces sp*. CFH 90414	Actinobacteria	-4
WP_163139709	*Arhodomonas sp.* KWT	Gammaproteobacteria	2
KQM84620	*Agromyces sp.* Leaf222	Actinobacteria	-4
WP_127795083	*Agromyces sp.* LHK192	Actinobacteria	-4
WP_170139737	*Cereibacter changlensis*	Alphaproteobacteria	7
WP_188511490	*Conyzicola nivalis*	Actinobacteria	-4
WP_052542113	*Cryobacterium roopkundense*	Actinobacteria	-4
WP_134519663	*Cryobacterium sp.* Hh14	Actinobacteria	-4
WP_062133603	*Demequina aestuarii*	Actinobacteria	-4
QKT02779	*Ectothiorhodospiraceae bacterium* 2226	Gammaproteobacteria	2
MBA1147819	*Ectothiorhodospiraceae bacterium* WFHF3C12	Gammaproteobacteria	2
WP_067609350	*Erythrobacter sp.* QSSC1-22B	Alphaproteobacteria	7
WP_161972385	*Glaciihabitans arcticus*	Actinobacteria	-4
TNF52830	*Gammaproteobacteria bacterium*	Gammaproteobacteria	2
WP_106210458	*Glaciihabitans tibetensis*	Actinobacteria	-4
TFH86654	*Halomonas azerbaijanica*	Gammaproteobacteria	7
WP_170840070	*Halomonas daqiaonensis*	Gammaproteobacteria	7
WP_133735568	*Halospina denitrificans*	Gammaproteobacteria	-4
WP_089685487	*Halomonas gudaonensis*	Gammaproteobacteria	7
WP_136546581	*Hydrocarboniclastica marina*	Gammaproteobacteria	7
WP_151441795	*Halomonas sp.* 3(2)	Gammaproteobacteria	7
WP_168014807	*Halomonas sp.* G5-11	Gammaproteobacteria	0
WP_163574662	*Halomonas sp.* JSM 104105	Gammaproteobacteria	7
WP_181421981	*Halomonas sp.* LBP4	Gammaproteobacteria	7
WP_163557478	*Halomonas sp.* NO4	Gammaproteobacteria	7

WP_181872794	*Halomonas montanilacus*	Gammaproteobacteria	7
WP_071694564	*Halomonas sp.* QHL1	Gammaproteobacteria	7
WP_175071359	*Halomonas taeanensis*	Gammaproteobacteria	14
WP_102586694	*Halomonas urumqiensis*	Gammaproteobacteria	7
WP_133634377	*Halomonas ventosae*	Gammaproteobacteria	7
WP_133538284	*Idiomarina aquatica*	Gammaproteobacteria	7
WP_006955969	*Idiomarina baltica*	Gammaproteobacteria	7
WP_110572772	*Idiomarina fontislapidosi*	Gammaproteobacteria	7
WP_198400859	*Idiomarina piscisalsi*	Gammaproteobacteria	7
WP_126783725	*Idiomarina seosinensis*	Gammaproteobacteria	7
WP_164976520	*Idiomarina sp.* 29L	Gammaproteobacteria	7
WP_007419415	*Idiomarina sp.* A28L	Gammaproteobacteria	7
WP_071463767	*Idiomarina sp.* MD25a	Gammaproteobacteria	7
NQZ15391	*Idiomarina sp.*	Gammaproteobacteria	7
NQZ03991	*Idiomarina sp.*	Gammaproteobacteria	7
KXS35797	*Idiomarina sp. T82-3*	Gammaproteobacteria	7
WP_198511823	*Idiomarina sp.* X4	Gammaproteobacteria	7
WP_135119181	*Leifsonia flava*	Actinobacteria	-4
WP_056167830	*Leifsonia sp.* Leaf325	Actinobacteria	-4
MBN9239507	*Leifsonia sp.*	Actinobacteria	-4
WP_027329460	*Marinimicrobium agarilyticum*	Gammaproteobacteria	14
WP_091958617	*Marinospirillum celere*	Gammaproteobacteria	2
WP_082334594	*Microbacterium chocolatum*	Actinobacteria	-4
WP_163780714	*Mycobacterium cookii*	Actinobacteria	-4
MBB5743985	*Microbacterium ginsengiterrae*	Actinobacteria	-4
WP_055579379	*Mycobacterium gordonae*	Actinobacteria	-4
WP_163749773	*Mycolicibacterium helvum*	Actinobacteria	-4
WP_168209470	*Mycobacterium helveticum*	Actinobacteria	-4
WP_203582382	*Microbacterium hibisci*	Actinobacteria	-4
WP_052682433	*Microbacterium ketosireducens*	Actinobacteria	-4
WP_123638291	*Marinimicrobium koreense*	Gammaproteobacteria	14
WP_185843127	*Microbacterium kyungheense*	Actinobacteria	-4
AZS35752	*Microbacterium lemovicicum*	Actinobacteria	-4
WP_052226828	*Microbacterium mangrovi*	Actinobacteria	-7
WP_165691513	*Mycobacterium numidiamassiliense*	Actinobacteria	-4
WP_120792055	*Mycobacterium paragordonae*	Actinobacteria	-4
WP_091706258	*Marinobacter persicus*	Gammaproteobacteria	2
WP_091492622	*Microbacterium pygmaeum*	Actinobacteria	-4
WP_166256481	*Marinobacter salicampi*	Gammaproteobacteria	7
WP_159498428	*Microbacterium sp.* 18062	Actinobacteria	-4
WP_165127206	*Microbacterium sp.* 4R-513	Actinobacteria	-4
WP_124292376	*Microbacterium sp.* ABRD_28	Actinobacteria	-4
WP_179559891	*Microbacterium sp.* AK009	Actinobacteria	-4

WP_163634198	*Microbacterium sp.* B35-04	Actinobacteria	-4
WP_163620054	*Microbacterium sp.* B35-30	Actinobacteria	-4
WP_133541699	*Microbacterium sp.* BK668	Actinobacteria	-4
WP_082497209	*Microbacterium sp.* Leaf288	Actinobacteria	-4
WP_036160751	*Marinimicrobium sp.* LS-A18	Gammaproteobacteria	14
WP_156686657	*Mycobacterium sp.* Marseille-P9652	Actinobacteria	-4
RUP06075	*Mycobacterium sp.*	Actinobacteria	0
WP_191170259	*Microbacterium helvum*	Actinobacteria	-4
WP_191764400	*Microbacterium sp.* Sa1CUA4	Actinobacteria	-4
WP_141939844	*Microbacterium sp.* SLBN-154	Actinobacteria	-4
KXS53631	*Marinobacter sp.* T13-3	Gammaproteobacteria	2
WP_135955549	*Marinobacter orientalis*	Gammaproteobacteria	14
WP_194396676	*Microbacterium sp.* WY121	Actinobacteria	-4
WP_052676815	*Microbacterium trichothecenolyticum*	Actinobacteria	-4
WP_083866257	*Microbacterium yannicii*	Actinobacteria	-4
WP_121658974	*Mycetocola zhadangensis*	Actinobacteria	-4
WP_158270294	*Mycetocola zhujimingii*	Actinobacteria	-4
WP_159084898	*Planctomonas deserti*	Actinobacteria	-4
WP_119515934	*Pusillimonas maritima*	Betaproteobacteria	-7
WP_034774458	*Pseudidiomarina salinarum*	Gammaproteobacteria	7
WP_150304946	*Pseudomonas saliphila*	Gammaproteobacteria	14
WP_185779599	*Pusillimonas sp.* 7-48	Betaproteobacteria	-7
WP_166126703	*Pusillimonas sp.* DMV24BSW_D	Betaproteobacteria	-7
WP_119515934	*Pusillimonas maritima*	Betaproteobacteria	-7
MBC7201962	*Pusillimonas sp.*	Betaproteobacteria	-7
WP_123659738	*Pusillimonas sp.* NJUB218	Betaproteobacteria	-7
WP_093969838	*Pusillimonas sp.* T2	Betaproteobacteria	-7
WP_187673991	*Protaetiibacter sp.* SSC-01	Actinobacteria	-4
MBN9023294	*Hyphomicrobiales bacterium*	Alphaproteobacteria	7
WP_205015307	*Rhodoligotrophos appendicifer*	Alphaproteobacteria	7
WP_037335575	*Salinisphaera hydrothermalis*	Gammaproteobacteria	-7
KEZ78150	*Salinisphaera hydrothermalis* C41B8	Gammaproteobacteria	-7
WP_006912426	*Salinisphaera shabanensis*	Gammaproteobacteria	-7
MBA2635521	*Sphingomonas sp.*	Alphaproteobacteria	7
WP_075148475	*Thauera chlorobenzoica*	Betaproteobacteria	-7
MBA2666381	*Trueperaceae bacterium*	Deinococci	-7
WP_159432998	*Thiohalospira halophila*	Gammaproteobacteria	2
SFD01659	*Thiohalospira halophila DSM* 15071	Gammaproteobacteria	2
WP_018949990	*Thioalkalivibrio sp.* ALMg11	Gammaproteobacteria	0
WP_019567925	*Thioalkalivibrio sp.* ALMg13-2	Gammaproteobacteria	0
WP_019594312	*Thioalkalivibrio sp.* ALM2T	Gammaproteobacteria	0
WP_026331574	*Thioalkalivibrio sp.* ALMg3	Gammaproteobacteria	0
WP_026304848	*Thioalkalivibrio sp.* AKL8	Gammaproteobacteria	0

WP_024326261	*Thioalkalivibrio sp.* AKL19	Gammaproteobacteria	0
WP_134083281	*Thiohalophilus thiocyanatoxydans*	Gammaproteobacteria	0
WP_026186278	*Thioalkalivibrio thiocyanodenitrificans*	Gammaproteobacteria	14
WP_047251462	*Thioalkalivibrio versutus*	Gammaproteobacteria	0
WP_164231063	*Wenzhouxiangella sp.* XN201	Gammaproteobacteria	0
WP_159340624	*Halomonas meridiana*	Gammaproteobacteria	14
WP_072324728	*Marinospirillum alkaliphilum*	Gammaproteobacteria	2
	**PadC-EALs**		
WP_052022258	*Actinotalea ferrariae*	Actinobacteria	-4
WP_114207983	*Acidisarcina polymorpha*	Acidobacteria	-5
WP_024476458	*Arthrobacter sp.* CAL618	Actinobacteria	-4
WP_119157670	*Aquabacterium tepidiphilum*	Betaproteobacteria	24
KAB2309365	*Betaproteobacteria bacterium* SCN2	Betaproteobacteria	-7
WP_081153798	*Colwellia beringensis*	Gammaproteobacteria	0
MBD5655562	*Candidatus Eremiobacteraeota bacterium*	Candidatus Eremiobacteria	0
WP_143870274	*Catenovulum sediminis*	Gammaproteobacteria	0
WP_081181501	*Colwellia sp.* PAMC 21821	Gammaproteobacteria	0
WP_056147085	*Duganella sp.* Leaf61	Betaproteobacteria	0
WP_175529082	*Granulicella pectinivorans*	Acidobacteria	-4
MBC7573121	*Herminiimonas sp.*	Betaproteobacteria	0
WP_158257126	*Kineococcus xinjiangensis*	Actinobacteria	-4
WP_171837799	*Lichenicola cladoniae*	Alphaproteobacteria	-4
WP_069331786	*Luteovulum johrii*	Alphaproteobacteria	-4
SNX71557	*Luteovulum ovatus*	Alphaproteobacteria	-4
WP_138326492	*Lichenicoccus roseus*	Alphaproteobacteria	-4
WP_111607365	*Marinomonas arctica*	Gammaproteobacteria	0
WP_060937607	*Mycolicibacterium chlorophenolicum*	Actinobacteria	-4
WP_053081353	*Mycolicibacterium chubuense*	Actinobacteria	-4
WP_099788017	*Massilia eurypsychrophila*	Betaproteobacteria	0
WP_178360910	*Mycolicibacterium hippocampi*	Actinobacteria	-4
WP_142550055	*Mycolicibacterium hodleri*	Actinobacteria	-4
WP_163720540	*Mycolicibacterium psychrotolerans*	Actinobacteria	-4
MBI5341653	*Mycolicibacterium rufum*	Actinobacteria	-4
WP_203301017	*Marinobacter sediminum*	Gammaproteobacteria	0
WP_111637363	*Marinomonas shanghaiensis*	Gammaproteobacteria	0
WP_051933242	*Massilia sp.* BSC265	Betaproteobacteria	0
WP_137146372	*Mycolicibacterium sp.* CR10	Actinobacteria	-4
WP_193687304	*Massilia sp.* LPB0304	Betaproteobacteria	0
MBC7454863	*Massilia sp.*	Betaproteobacteria	-4
MBC7453434	*Massilia sp.*	Betaproteobacteria	0
WP_171086708	*Massilia sp.* ML15P13	Betaproteobacteria	0
WP_168710040	*Massilia sp.* Mn16-1_5	Betaproteobacteria	0
WP_056547734	*Mycobacterium sp.* Root135	Actinobacteria	-4

WP_169469313	*Massilia sp.* RP-1-19	Betaproteobacteria	-4
QOY95134	*Massilia sp.* UMI-21	Betaproteobacteria	0
WP_180540017	*Nevskia soli*	Gammaproteobacteria	-7
WP_124797577	*Nakamurella sp.* s14-144	Actinobacteria	-4
NDP59305	*Oxalobacteraceae bacterium*	Betaproteobacteria	0
WP_052713144	*Pseudoalteromonas rubra*	Gammaproteobacteria	0
WP_185745544	*Pseudoalteromonas sp.* J010	Gammaproteobacteria	0
WP_076485451	*Rhodobacter aestuarii*	Alphaproteobacteria	7
WP_150045626	*Rhodovastum atsumiense*	Alphaproteobacteria	-4
WP_101342037	*Rhodobacter azotoformans*	Alphaproteobacteria	-4
WP_140884289	*Roseomonas nepalensis*	Alphaproteobacteria	-2
WP_145110360	*Rhodobacter sediminicola*	Alphaproteobacteria	-4
EKX59214	*Rhodobacter sp.* AKP1	Alphaproteobacteria	-4
WP_198402499	*Rhodobacter sp.* CZR27	Alphaproteobacteria	-4
WP_097082434	*Rhodobacter sp.* JA431	Alphaproteobacteria	7
WP_081532018	*Rhodovulum sp.* P5	Alphaproteobacteria	7
WP_168746016	*Luteovulum sphaeroides*	Alphaproteobacteria	-4
RDS94692	*Rhodobacter sphaeroides f. sp. denitrificans*	Alphaproteobacteria	-4
WP_104507055	*Rhodoblastus sphagnicola*	Alphaproteobacteria	-4
WP_110807469	*Rhodobacter viridis*	Alphaproteobacteria	7
MBA2512727	*Solirubrobacterales bacterium*	Actinobacteria	-4
SEO67024	*Salinihabitans flavidus*	Alphaproteobacteria	0
WP_149819107	*Salinarimonas sp.* BN140002	Alphaproteobacteria	-4
RZA35242	*Xanthomonadaceae bacterium*	Gammaproteobacteria	0
WP_100368535	*Yoonia maricola*	Alphaproteobacteria	14

Supp. Table 4: List of sequences removed from the compilation of PadCs and PadC-EALs *due to their annotation being changed to "obsolete"*

WP_116082607	WP_204980845	WP_192633537	WP_141885470
WP_114574856	WP_018173226	WP_159841378	WP_026330496
WP_173087016	NPD70263	NCA88702	MBM0930487
WP_184425893	MBC7374002	MBJ7339020	NVK71981
ABP73126			

Supp. Table 5: Representative bacteriophytochrome architectures with more than one output domain per organism*, as depicted in Figure 2. HK = Histidine kinase; RR = response regulator; HWE-HK = HWE histidine kinase; DGC = diguanylate cyclase; SpoIIE = Bacillus subtilis stage II sporulation protein E.*

Uniprot acc. No	NCBI acc. No	Organism	effectors
A0A0K1H4P2	AKT76079	*Chroomonas sp. ROZZ_2007332*	Outgroup
A0A3E0WQH3	WP_116303044	*Alkalilimnicola ehrlichii*	HK
A0A5B9W4T4	WP_148595089	*Aquisphaera giovannonii*	HK
H0SNP3	WP_009030726	*Bradyrhizobium sp.* ORS 375	HK
A0A2U8W9H7	WP_109891510	*Methylobacterium durans*	HK
A0A346YU76	WP_118852258	*Neorhizobium sp.* SOG26	HK
A0A5M6INW8	WP_150043017	*Rhodovastum atsumiense*	HK
B3Q8U2	WP_012496490	*Rhodopseudomonas palustris*	HK
A0A5B9W750	WP_168221974	*Aquisphaera giovannonii*	HK + RR
A0A2U8WCF2	WP_109894975	*Methylobacterium durans*	HK + RR
H0SIV0	WP_009029074	*Bradyrhizobium sp.* ORS 375	HWE-HK
A0A2N5DSC1	WP_101716079	*Caulobacter zeae*	HWE-HK
A0A5R9J5W9	WP_138327916	*Lichenicoccus roseus*	HWE-HK
A0A2U8W941	WP_109892731	*Methylobacterium durans*	HWE-HK
A0A2R4GQ37	WP_107354401	*Rhodopseudomonas palustris*	HWE-HK
A0A346YM92	WP_118849843	*Neorhizobium sp.* SOG26	HWE-HK + RR
B3Q7C0	WP_012495153	*Rhodopseudomonas palustris*	PAS
A0A0Q2LIL1	KQH76073	*Mycobacterium gordonae*	SpoIIE
	WP_011629241	*Alkalilimnicola ehrlichii*	DGC
A0A0Q2R111	WP_055579379	*Mycobacterium gordonae*	DGC
A0A2N5DPH7	WP_101717170	*Caulobacter zeae*	HK2
H0SQA1	WP_009031270	*Bradyrhizobium sp.* ORS 375	PAS
A0A5M6IX79	WP_150040088	*Rhodovastum atsumiense*	PAS
	WP_138326492	*Lichenicoccus roseus*	DGC + EAL
	WP_150045626	*Rhodovastum atsumiense*	DGC + EAL

Supp. Table 6: Normalised DGC activity at 200 µM GTP. *Specific activity per dimer under non-actinic and actinic conditions, determined from four timepoints each. The error indicator represents the SE of the estimate from the linear regression of fitting the initial velocities. The dynamic range equals the fold upregulation; values lower than 1 indicate red light-induced downregulation of enzymatic activity.*

PadC	Linker	Activity Dark		Activity Light		Dynamic Range
*Is*PadC^{+7}	+7	0.8	± 0.1	34.2	± 0.4	44
*Ma*PadC^{+14}	+14	2.1	± 0.3	15.4	± 0.8	7.5
*Ts*PadC^{0}	0	8.0	± 0.7	52	± 3	6.5
*Cc*PadC^{+7}	+7	0.42	± 0.02	18.7	± 1.6	44
*Ra*PadC^{+7}	+7	0.07	± 0.02	0.6	± 0.2	8.9
*Ps*PadC^{-7}	-7	0.044	± 0.005	0.72	± 0.04	16
*As*PadC^{-4}	-4	1.91	± 0.07	5.2	± 0.2	2.7
*Mt*PadC^{-4}	-4	0.169	± 0.006	2.2	± 0.1	13
*Mm*PadC^{-7}	-7	1.50	± 0.05	3.3	± 0.1	2.2
*Hd*PadC^{-4}	-4	11.1	± 0.8	18.9	± 1.4	1.7
*Mp*PadC^{+2}	+2	2.9	± 0.2	2.45	± 0.02	0.9
*Ms*PadC^{+2}	+2	15.3	± 1.2	5.9	± 0.4	0.4
*Th*PadC^{+2}	+2	0.088	± 0.005	0.006	± 0.0001	0.1
*Ars*PadC^{+2}	+2	No c-di-GMP production detected				

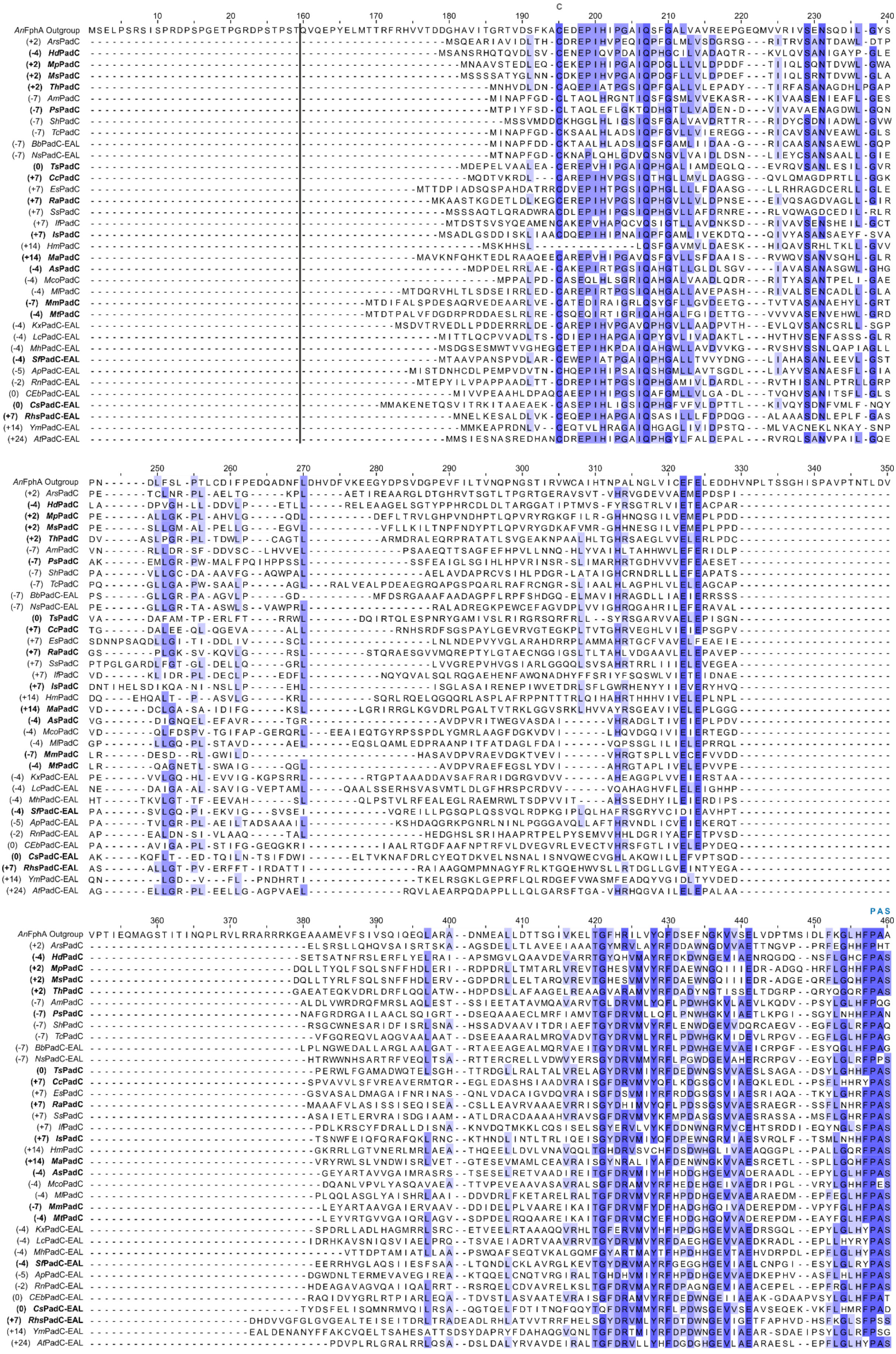

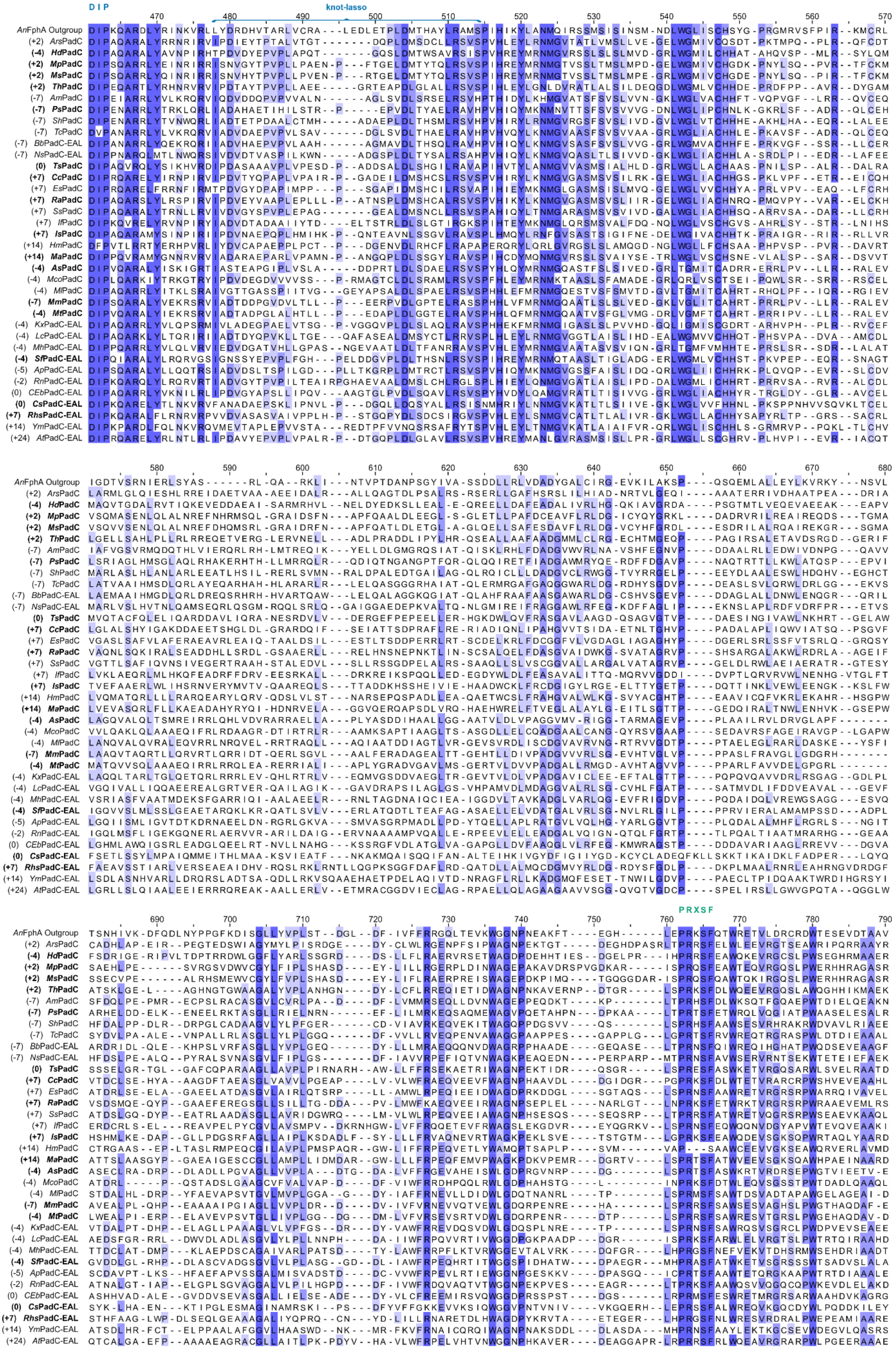
DIP
knot-lasso
PRXSF

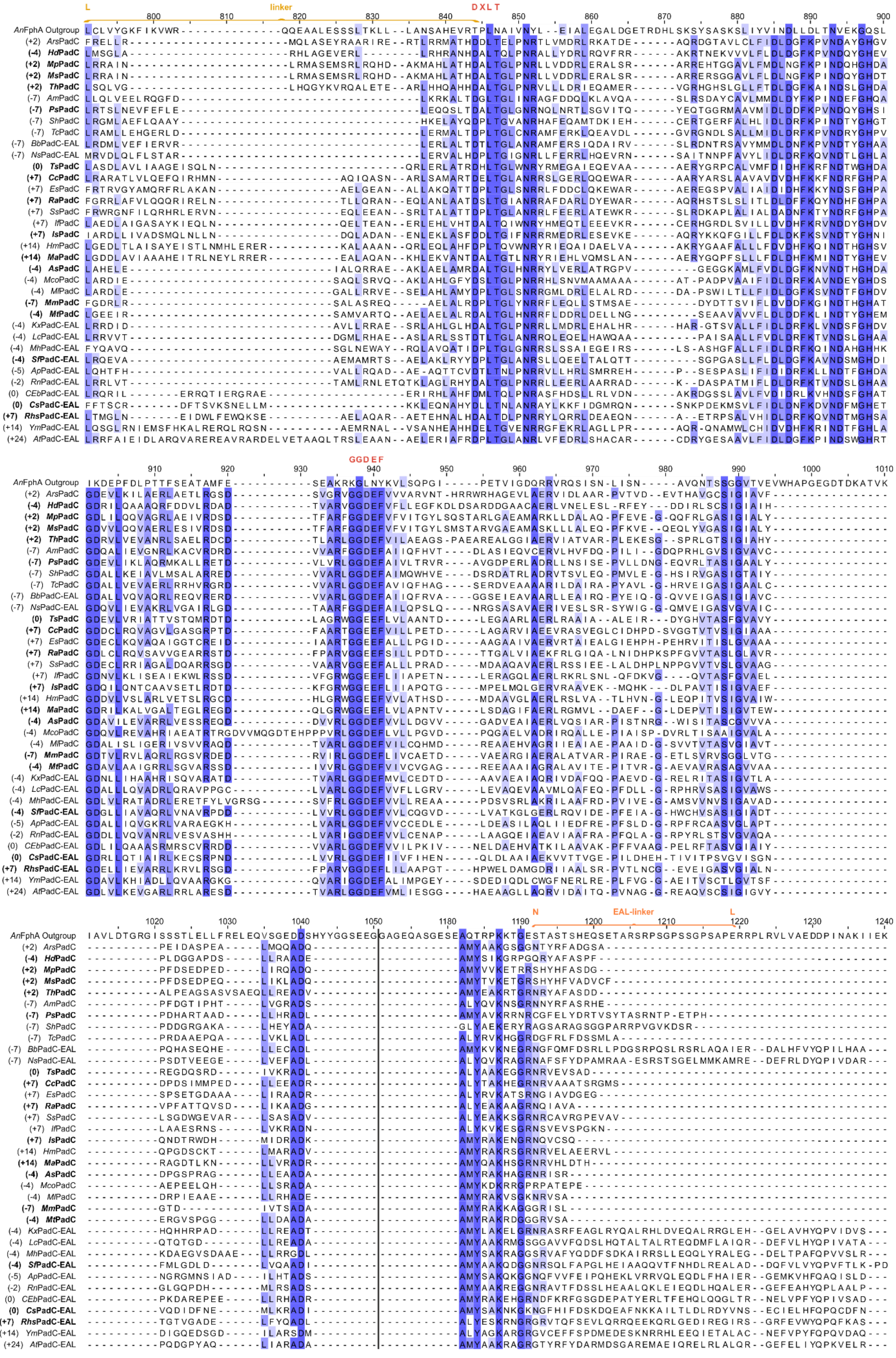
L
linker
DXLT
GGDEF
N
EAL-linker
L

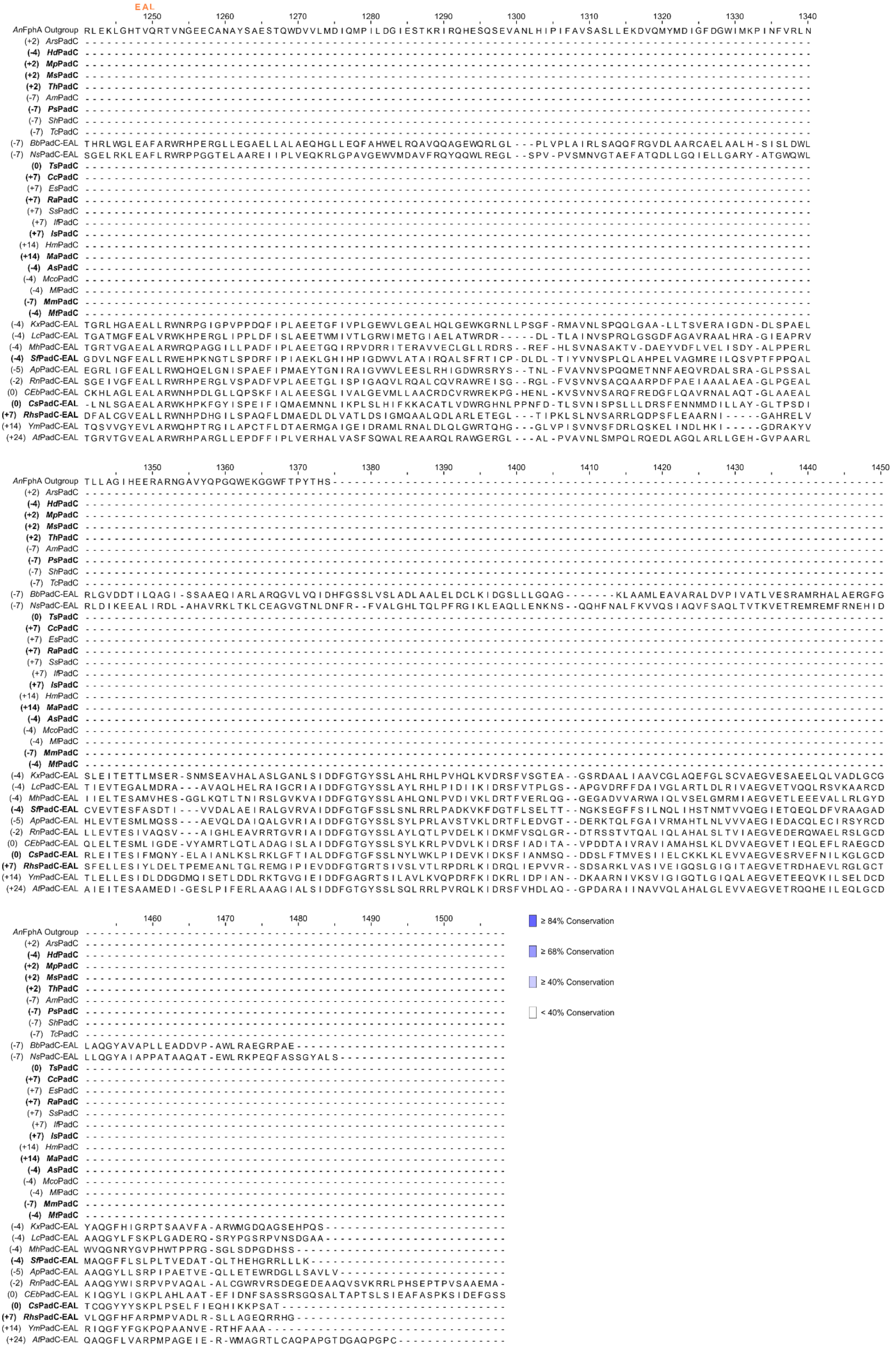
EAL
≥ 84% Conservation
≥ 68% Conservation
≥ 40% Conservation
< 40% Conservation

Supp. Fig. 1: Complete Alignment of PadC and PadC-EAL sequences represented in Figure 2. *Essential motifs and sequence elements are provided above the alignment: The chromophore-binding cysteine in the NTS (grey), the PASDIP motif and the knot-lasso in the GAF domain (blue), the PRXSF motif in the PHY-tongue (green), the linker element as defined in this paper (yellow) ending with the DXLT motif that defines the beginning of the GGDEF domain (red), the GGDEF motif (red), and the EAL-linker and EAL motif (orange).*

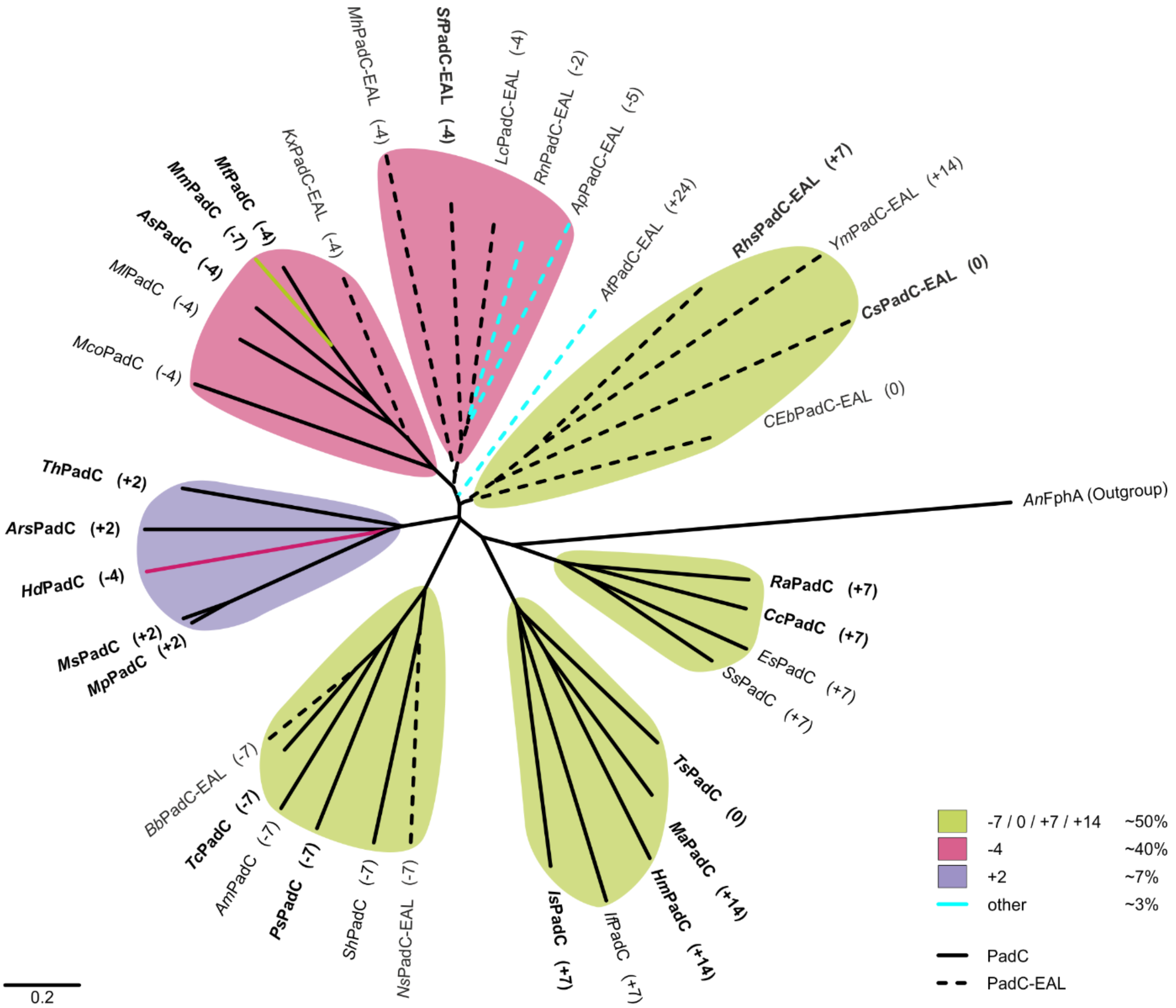

Supp. Fig. 2: Distance Tree of representative PadCs and PadC-EALs*, as depicted in the bootstrapped tree in Figure 3. Bold entries represent investigated homologs. Homologs strongly cluster according to linker length.*

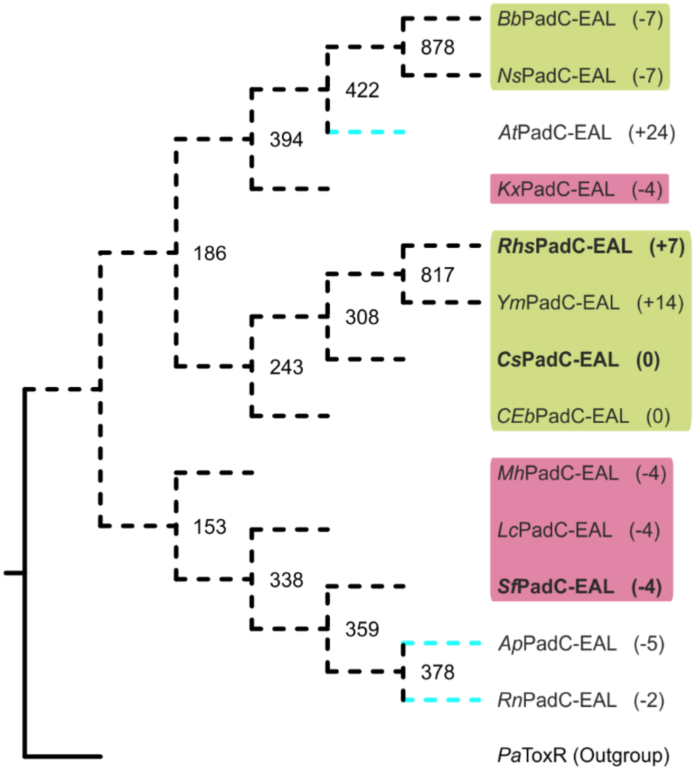

Supp. Fig. 3: Bootstrap tree of the EAL domains *from all PadC-EALs shown in Figure 3 and Supp. Figure 1. The alignment was cut off in position 1191, and the stand-alone EAL protein ToxR/RegA from Pseudomonas aeruginosa (NCBI accession number: AAG04096) introduced as outgroup. The bootstrap tree was subsequently created as described in Materials and Methods. No cluster of EAL-domains is significant as such.*

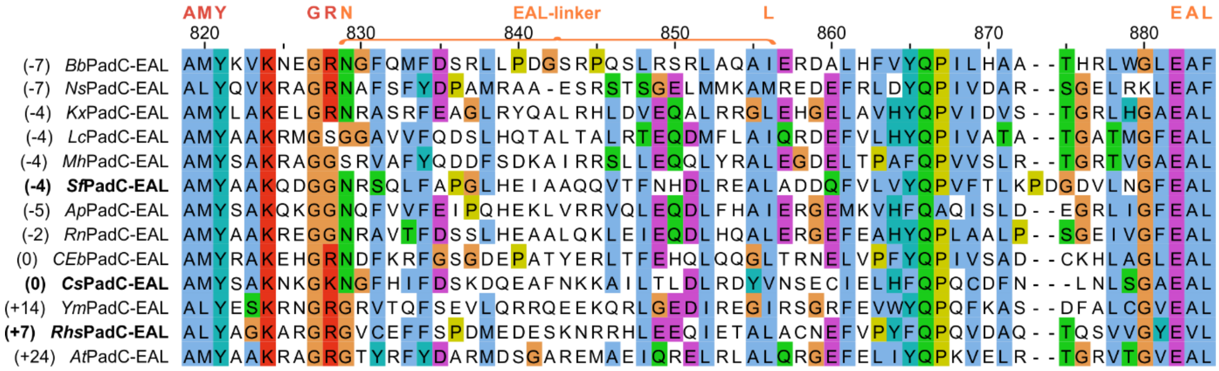

Supp. Fig. 4: Alignment of the EAL-linker for all PadC-EALs *listed in Supp. Table 1, coloured by Jalview option "Clustalx". A high level of conservation is observed for several residues across all homolgs; including BbPadC-EAL^{-7} and NsPadC-EAL^{-7}, two homologs that cluster with PadCs^{-7} rather than other PadC-EALs in phylogenetic distance trees.*

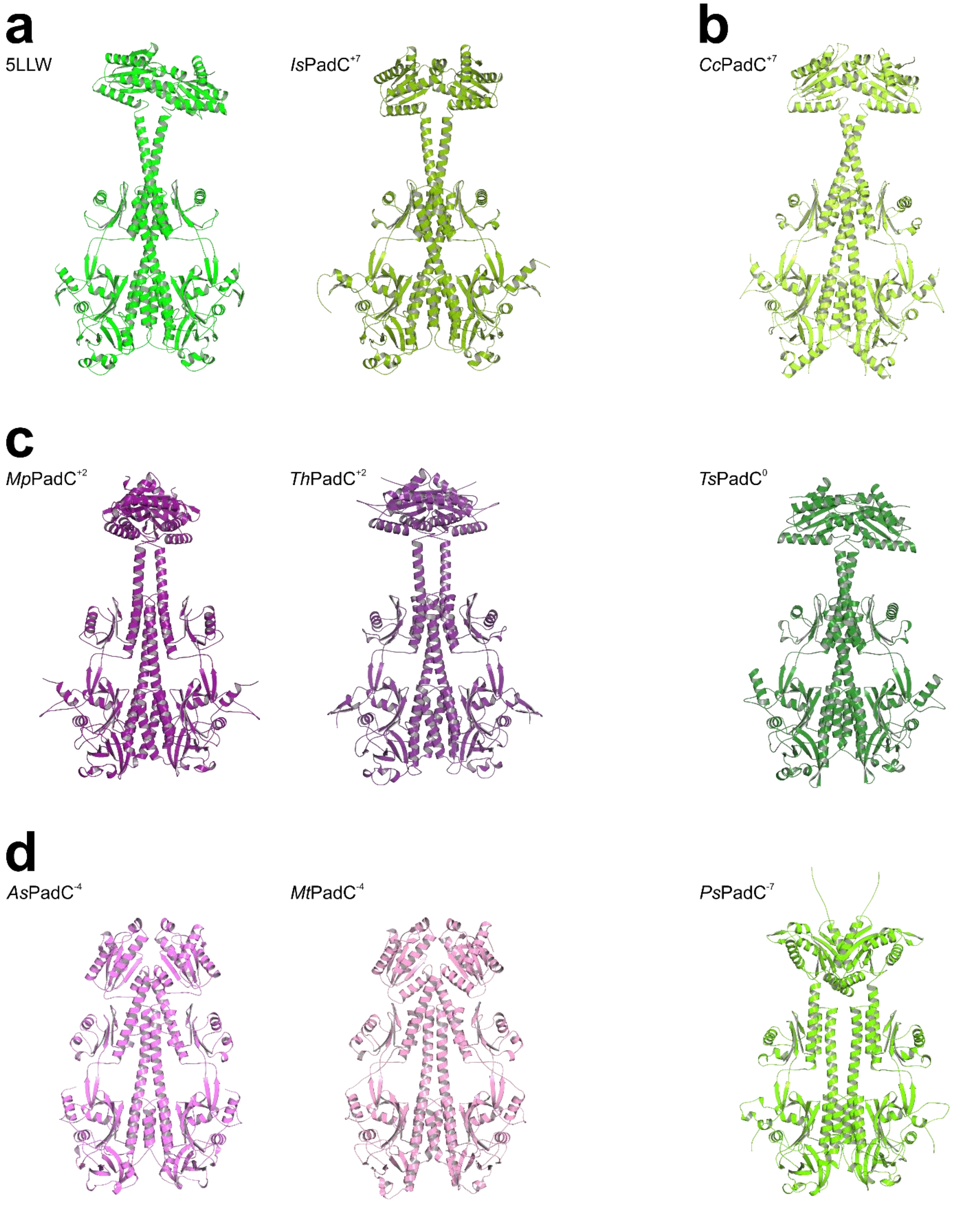

Supp. Fig. 5: Representative AlphaFold2 predictions of various PadCs. (a) *Comparison of the AlphaFold2-predicted model of IsPadC^{+7} to the IsPadC^{+7} crystal structure (pdb 5LLW).* ***(b – d)*** *AlphaFold2 predictions based on representative sequences of PadCs with linker lengths divisible by seven, the PadC^{+2} group, and the PadC^{-4} branch, respectively.*

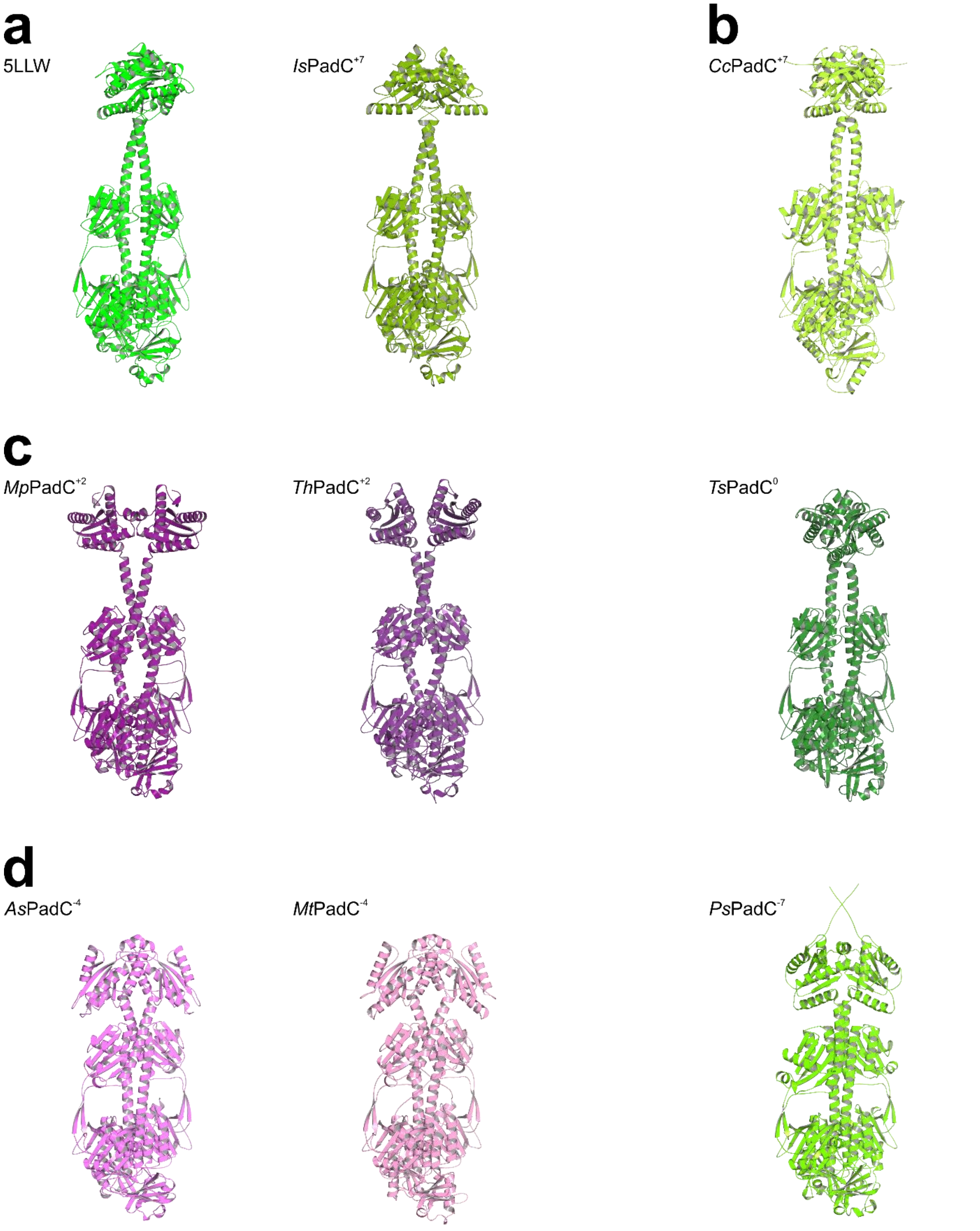

Supp. Fig. 6: Rotated view of representative AlphaFold2 predictions of various PadCs. (a – d) *AlphaFold2 predictions based on representative sequences of 5LLW and IsPadC*$^{+7}$*, PadCs with linker lengths divisible by seven, the PadC*$^{+2}$ *family, and the PadC*$^{-4}$ *branch, respectively.*

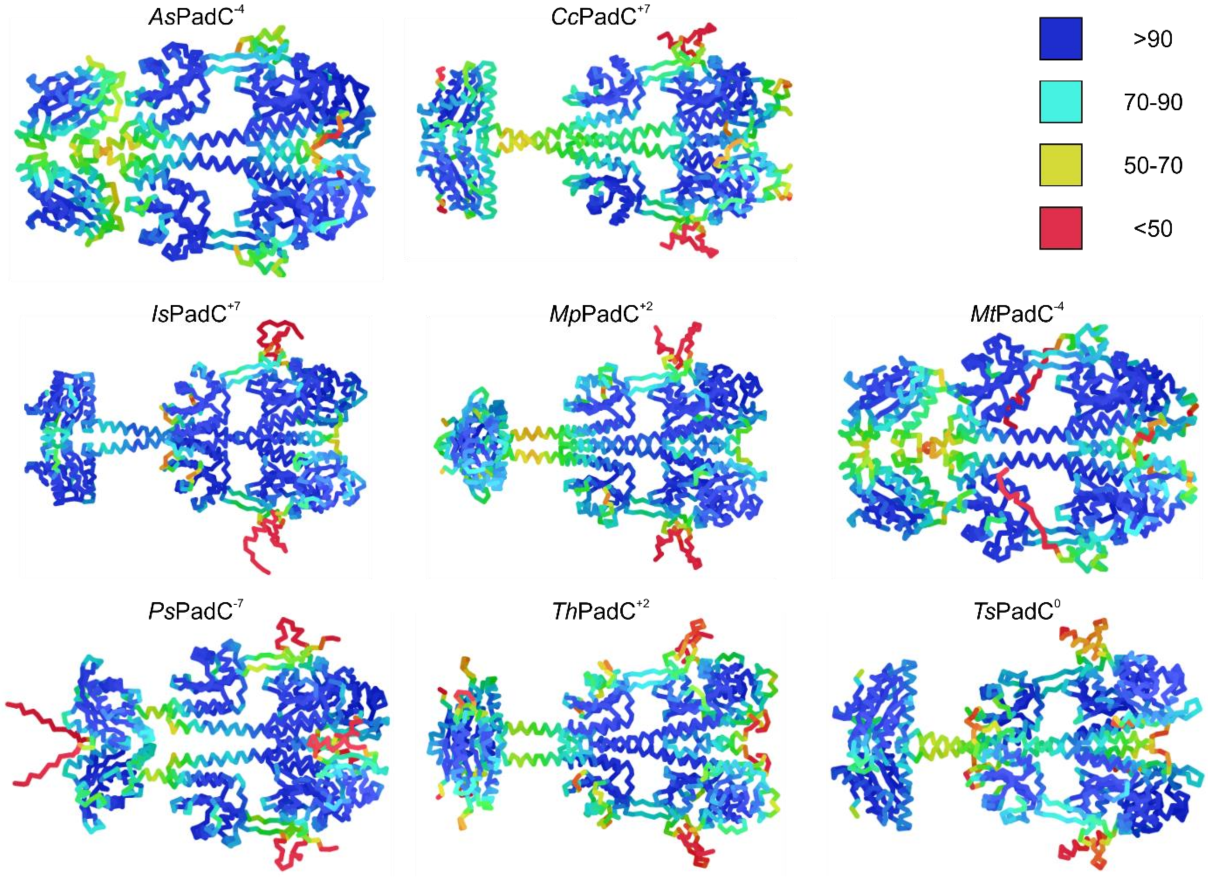

Supp. Fig. 7: plDDT scores mapped onto structures of all AlphaFold2 predictions *presented in Figure 8. High plDDT (predicted local distance difference test) scores indicate high confidence in local correctness of the prediction, and are indicated by blue colour. Loop areas, the NTS, and in many cases the linker element display lower levels of confidence in the prediction.*

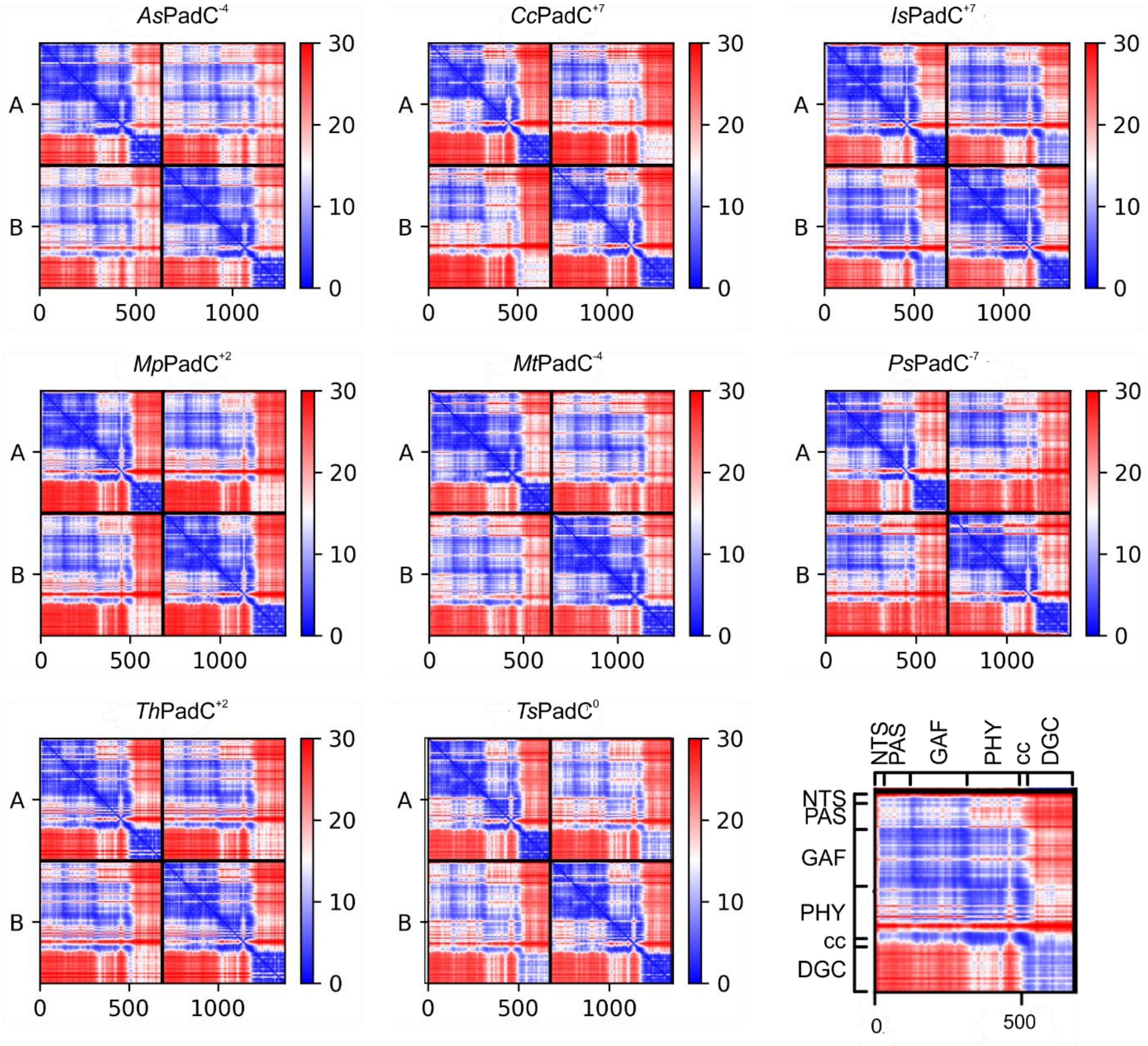

***Supp. Fig. 8: PAE score of AlphaFold2 predicted models** presented in Figure 8. PAE = Predicted Aligned Error, blue indicates a low PAE score indicates higher pairwise confidence in inter-domain interactions, within and between protomers (A and B).*

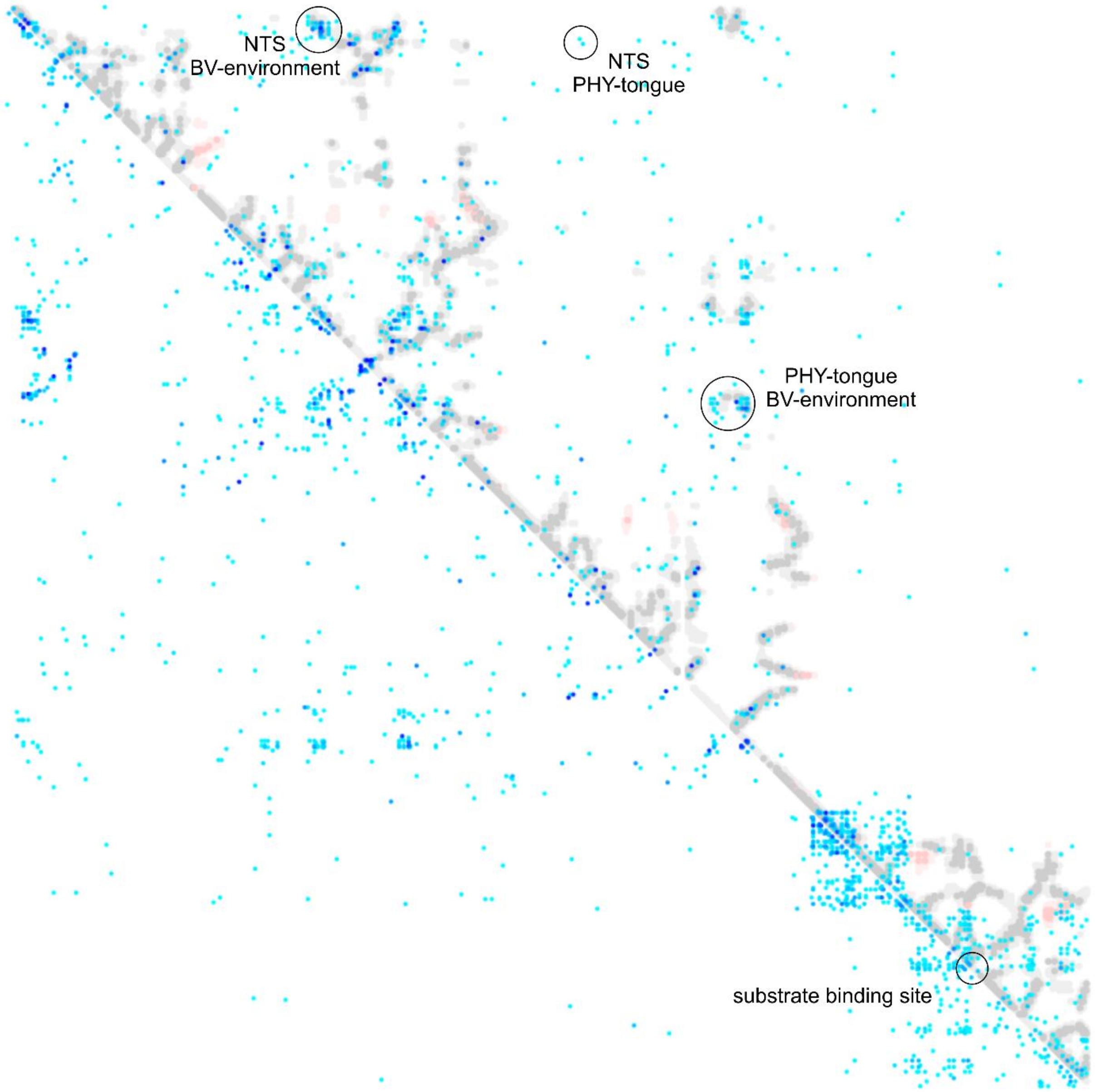

Supp. Fig. 9: GREMLIN results overlaid on 5LLX *(IsPadC^{+7} co-crystallized with substrate). Grey dots indicate PDB residue contacts, blue dots represent coevolution (darker tones of blue correspond to higher coevolution strength).*
